# A new nanocomposite forward osmosis membrane custom-designed for treating shale gas wastewater

**DOI:** 10.1038/srep14530

**Published:** 2015-09-29

**Authors:** Detao Qin, Zhaoyang Liu, Darren Delai Sun, Xiaoxiao Song, Hongwei Bai

**Affiliations:** 1 Energy Research Institute @ NTU, Interdisciplinary Graduate School, Nanyang Technological University, 639798, Singapore; 2 Qatar Environment and Energy Research Institute (QEERI), HBKU, Qatar Foundation, PO Box 5825, Doha, Qatar; 3 School of Civil and Environmental Engineering, Nanyang Technological University, 639798, Singapore; 4 Energy Research Institute @ NTU, Nanyang Technological University, 639798, Singapore

## Abstract

Managing the wastewater discharged from oil and shale gas fields is a big challenge, because this kind of wastewater is normally polluted by high contents of both oils and salts. Conventional pressure-driven membranes experience little success for treating this wastewater because of either severe membrane fouling or incapability of desalination. In this study, we designed a new nanocomposite forward osmosis (FO) membrane for accomplishing simultaneous oil/water separation and desalination. This nanocomposite FO membrane is composed of an oil-repelling and salt-rejecting hydrogel selective layer on top of a graphene oxide (GO) nanosheets infused polymeric support layer. The hydrogel selective layer demonstrates strong underwater oleophobicity that leads to superior anti-fouling capability under various oil/water emulsions, and the infused GO in support layer can significantly mitigate internal concentration polarization (ICP) through reducing FO membrane structural parameter by as much as 20%. Compared with commercial FO membrane, this new FO membrane demonstrates more than three times higher water flux, higher removals for oil and salts (>99.9% for oil and >99.7% for multivalent ions) and significantly lower fouling tendency when investigated with simulated shale gas wastewater. These combined merits will endorse this new FO membrane with wide applications in treating highly saline and oily wastewaters.

The shale gas boom has been one of the biggest energy topics worldwide in recent years^1^,^2^. Specifically, annual production volume of shale gas is expected to grow more than threefold over the next decade in the United States of America^3^. Horizontal drilling and hydraulic fracturing for exploiting natural gas from unconventional shale formations produce large quantities of wastewaters with unique characteristics of high salinity and oil contents^4^,^5^, which generates unprecedented challenge for the selection of appropriate water treatment technologies to prevent environmental and health damages from disposing these wastewaters^6^,^7^,^8^. Therefore, the development of feasible and practical technologies with the genuine capability of concurrently removing both oils and salts from these produced wastewaters is highly desirable for shale gas industries.

Membrane technology has been considered as a promising approach for treating shale gas produced wastewater with smaller footprint and less equipment investment^9^,^10^. Reverse osmosis (RO) membranes are normally employed to treat high-salinity water, such as seawater. Operated under high hydraulic pressure, RO membrane technology is typically associated with two intrinsic issues: (1) high operational energy consumption, and (2) irreversible membrane fouling and subsequent flux decline especially when treating water with high content of organic foulants, such as oil and grease^11^,^12^. As a result, there is few successful story with RO membranes for treating shale gas produced wastewater. In contrast to reverse osmosis, forward osmosis (FO) membrane process is driven by an osmotic gradient between the feed solution and the draw solution on the permeate side that has a higher osmotic pressure than the feed solution^13^,^14^,^15^. Because it is driven by osmotic pressure rather than hydraulic pressure, FO has a relatively low propensity for irreversible fouling of membranes^16^,^17^,^18^. This low fouling propensity can improve the overall FO process efficiency by reducing the pretreatment requirements for produced water and their associated energies and costs^19^,^20^. Recently, a pilot-scale operation of FO system using ammonia-carbon dioxide draw solution demonstrated the promising potential for desalinating shale gas produced water^21^,^22^. However, the current development of FO membrane still lags far behind in meeting the special requirements for treating shale gas wastewaters. The only commercially available FO membrane (HTI, USA) has intrinsic drawbacks with: (1) high membrane fouling propensity because of the relatively hydrophobic property of its membrane material (cellulose triacetate, whose water contact angle is ~87°, as shown in this study); and (2) low water flux because of its unfavorable membrane structure (low porosity and high tortuosity, which cause severe internal concentration polarization)^23^,^24^,^25^. Therefore, it is in high demand for FO membranes that are custom-designed for treating shale gas produced wastewater with the merits of ultralow membrane fouling and high water flux.

Recently, there were some interesting studies that made use of superwetting mechanisms to reduce membrane fouling or enhance water flux for separating salinity-free oil/water mixtures^26^,^27^,^28^,^29^. Specifically, the study of underwater superoleophobic meshes or membranes for oil/water separation has aroused considerable attention^30^,^31^,^32^. Typically, the surface of mesh or membrane was coated with superhydrophilic hydrogel (such as polyacrylamide^33^, polyacrylic acid^34^, or polyvinyl alcohol^35^) or ceramic (such as zeolite^36^ or TiO_2_^37^). And these meshes or membranes showed ultralow oil fouling tendency and high water flux when operated under external pressure. However, there was no study showing these pressure-driven meshes or membranes used for oil/water separation are capable of removing salts from water. Meanwhile, our previous studies showed an interesting phenomenon that using nanomaterial (such as electrospun nanofibers) as support layer of FO membrane can effectively improve the membrane structure, which results in reduced internal concentration polarization (ICP, see the concept of ICP in Supplementary Information (SI)) and significantly enhanced water flux^38^,^39^. However, the low production rate and high cost of electrospun nanofibers hinder the practical applications of these FO membranes.

Here, we report a new nanocomposite FO membrane that is custom-designed for treating shale gas produced wastewaters with combined merits of ultralow membrane fouling, high water flux and high salt rejection. This nanocomposite FO membrane consists of a highly underwater oleophobic hydrogel selective layer on top of a nanomaterial infused polymeric support layer. Herein, the hydrated and chemically-crosslinked polyvinyl alcohol (PVA) hydrogel was chosen as the selective layer considering its unique properties of oil-repellency and salt-rejection. And GO nanosheet was chosen to infuse into the support layer because this GO nanosheet can bring great benefit for optimizing the pore structures of the support layer and thus significantly enhancing FO water flux. In contrast to previously reported electrospun nanofibers, this GO infused polymeric support layer was synthesized by established phase inversion technique^40^ that is ready for commercial scale up. To our best knowledge, this is the first report on a FO membrane with integrated properties of oil repellency, salt rejection and high water flux that targets at shale gas produced wastewaters.

## Methods

### Synthesis of GO

GO nanosheets were prepared via a modified Hummer’s method^41^,^42^. The relevant details of experiment are provided in SI.

### Casting GO infused polymeric support layer

Note that weight fraction (wt%) refers to the proportion of entire dope solution (*i.e.* GO + polymer + additive + solvent). As-synthesized graphite oxide was sonicated in N, N-dimethylformamide (DMF) at a certain weight fraction (*e.g.* 0.2 wt%) to obtain a homogenous graphene oxide dispersion. 1 wt% polyvinyl pyrrolidone (PVP, Mw 55 kDa, additive) was dissolved in the GO solution under mechanical stirring at room temperature. Only after PVP was totally dissolved would 15 wt% polyethersulfone (PES, Mw 53 kDa) be added. Then the mixture was stirred at 60 °C for 24 hours to obtain the homogenous nanocomposite dope solution. A stainless steel knife (elcometer) with gate height set as 150 μm was applied to cast the dope solution into a thin film on a clean glass plate. The cast film was immediately immersed into a coagulation bath (DI water, 20 °C) for initializing phase inversion. 20 min later, the as-prepared GO infused polymeric support layer was carefully peeled off from the glass plate and annealed in water bath at 90 °C before stocked in 4 °C DI water. Pristine polymeric support layer was fabricated as the control group using the same method except that the GO wt% is zero. The pure water permeability and the rejection of polyethylene oxide (PEO, Mw 300 kDa, 200 mg/L) were tested for both pristine and GO infused polymeric support layers under external pressure of 1.0 bar.

### Coating chemically-crosslinked hydrogel selective layer

The hydrogel selective layer was synthesized through dip-coating crosslinked PVA on top surface of as-prepared support layer. Firstly, PVA (99+% hydrolyzed, Mw 89 ~ 98 kDa) powder was dissolved in DI water at 90 °C under mechanical stirring to obtain 0.25 wt% aqueous solution. Secondly, glutaraldehyde (GA, 25 wt% aqueous solution) was added into the PVA aqueous solution in precise amount corresponding to the theoretical crosslinking degree^43^ of 30%. The crosslinking reaction was heated at 60 °C for 15 min with 1 wt% 2M H_2_SO_4_ as the catalyst to obtain the crosslinked hydrogel solution. Thirdly, the as-prepared support layers were dip-coated in the crosslinked hydrogel solution with only top surface in contact with coating solution. Finally, after draining off the excess coating solution, the nascent FO membranes were dried at room temperature and further cured in oven at 100 °C for 10 min before stocked in 4 °C DI water.

### Determination of FO water flux (*J*_*V*_) and reverse salt flux (*J*_*S*_)

HTI FO membrane (cellulose triacetate, woven) was employed as the comparison throughout all performance tests. A custom-built FO system equipped with cross-flow cell was used to determine membrane performance (Figure S2, SI). Membrane orientation was fixed as selective layer facing feed solution (FO mode). And the draw solution refers to 1.5 M Na_2_SO_4_ (500 ml) except the determination of FO membrane structure parameter (0.5 M Na_2_SO_4_). Other details about the determination of *J*_*V*_ and *J*_*S*_ are discussed in SI.

### Determination of FO membrane structural parameter (*S*)

FO membrane structural parameter (*S* value) is determined by ICP modelling as expressed in equation (1)^44^,^45^:
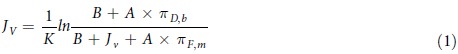
where *J*_*V*_ is FO water flux, *π*_*D,b*_ is osmotic pressure of draw solution bulk, *π*_*F,m*_ is osmotic pressure of feed solution at membrane surface, *A* is intrinsic water permeability of FO membrane, *B* is solute permeability of selective layer, and *K* is solute resistivity. Specifically, *B* and *K* can be determined by equation (2–3)^46^,^47^:
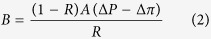

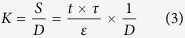
where *R* is solute rejection under RO mode, Δ*P* is the applied pressure, Δ*π* is osmotic pressure difference across the membrane, *D* is the diffusion coefficient of draw solute, and *S* is FO membrane structure parameter, which represents the average distance a draw solute molecule must travel when diffusing through the support layer^48^; *t, τ* and *ε* are membrane thickness, tortuosity and porosity, respectively.

In addition, intrinsic water permeability (*A*) and solute rejection (*R*) of FO membranes were determined by a lab-scale cross-flow RO test unit (Sterlitech, effective area 33.58 cm^2^).

### Preparation of saline oil-in-water emulsions

Vegetable oil and petroleum oils with different carbon numbers, namely *n*-hexane, 2,2,4-trimethylpentane (iso-octane), isopar-G, *n*-hexadecane and mineral oil were tested (see further information of these oils in Supplementary Experimental Details 2.3). The oil concentrations of emulsions were ranged from 2.5 to 100 g/L (g oil/L water). Triton X-100 was used as surfactant with the surfactant/oil ratios varying from 0.0 to 0.2. In order to simulate shale gas wastewater, hexadecane-in-water emulsions of different salinity (0 ~ 256 g/L total dissolved salts) were prepared according to the following procedure. Firstly, NaCl, MgSO_4_ and Al_2_(SO_4_)_3_ were dissolved in DI water (Millipore ultrapure water, 18 MΩ cm) with molar concentration 1:1:1. Secondly, surfactant and oil were added into the salt solution sequentially under mechanical stirring. Thirdly, the mixture was sonicated under 100 W at 20 °C for 3 hours to obtain a homogenous milky emulsion. Fresh emulsions were immediately used in the subsequent fouling tests.

### Evaluation of membrane fouling-resistance

For any particular feed solution, *J*_*V*_ reduction resulted from oil-fouling was reported as the average based upon parallel testing results of three pieces of membrane. For a particular piece of membrane, the testing consisted of a “baseline running” followed by a “oil-fouling running”, with separate batch of 500 ml 1.5 M Na_2_SO_4_ used as draw solution for each running. The protocol of testing is further elaborated as follows. Firstly, DI water (500 ml) was used as the feed solution to record a *J*_*V*_ baseline of 440 min, wherein the *J*_*V*_ value would drop gradually due to the osmotic dilution of draw solution. Secondly, “oil-fouling running” was performed in three sequential stages: “precondition” (40 min), “oil-fouling” (360 min) and “post-cleaning” (40 min). In “precondition” stage, the feed solution is still DI water. Oil-in-water emulsion was used as feed solution from 41^th ^min to 400^th ^min to study membrane fouling. After that, the membrane was *in situ* washed three times through flushing DI water in the feed side. Data recording was stopped during cleaning and resumed for another 40 min (designated as 401^th^ min to 440^th^ min) wherein DI water was reused as feed solution to investigate flux restoration. Note that in the feed tank the returning tubing tip of concentrate was placed 3 cm higher than water level in order to generate sufficient hydraulic mixing of feed solution. This setting of tubing can ensure membrane to confront the oil concentration truly as high as designated through eliminating any stratification of oil/water mixture during testing period, thus designed as the worst-scenario with respect to membrane fouling (see Supplementary Experimental Details 2.4).

Average flux reduction ratio (FRR), which indicates the loss of membrane permeability due to additional resistance induced by fouling for water molecule to overcome when permeating through membrane, was calculated for both “oil-fouling stage” and “post-cleaning stage” according to the following equations (4, 5):
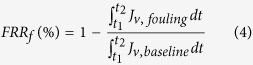

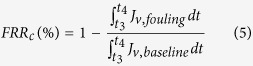
where *t*_*1*_ is 41^th ^min, *t*_*2*_ is 400^th ^min, *t*_*3*_ is 401^th^ min, *t*_*4*_ is 440^th^ min; *J*_*V,baseline*_ and *J*_*V,fouling*_ are *J*_***V***_ values of “baseline running” and “oil-fouling running”, respectively; *FRR*_*f*_ and *FRR*_*c*_ are flux reduction ratios of “oil-fouling stage” and “post-cleaning stage”, respectively. Note 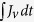 bears the physical meaning of FO throughput during a given period for 1 m^2^ membrane (L m^−2^).

### Simultaneous removal ratios

Simultaneous removal ratios of oil and salts by FO process were determined by the following equation (6):
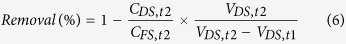
where *t*_*1*_ is 41^th^ min, *t*_*2*_ is 400^th ^min, *C*_*DS,t2*_ is the concentration in draw solution at *t*_*2*_, *C*_*FS,t2*_ is the concentration in feed solution at *t*_*2*_, *V*_*DS,t1*_ and *V*_*DS, t2*_ are volume of draw solution at *t*_*1*_ and *t*_*2*_, respectively. Note that the calculation of removal ratios by FO process should consider the dilution of permeates in draw solution.

### Characterization

The details of characterization are discussed in SI.

## Results

### Synthesis of oil-repelling and salt-rejecting nanocomposite FO membrane

**Figure 1:**
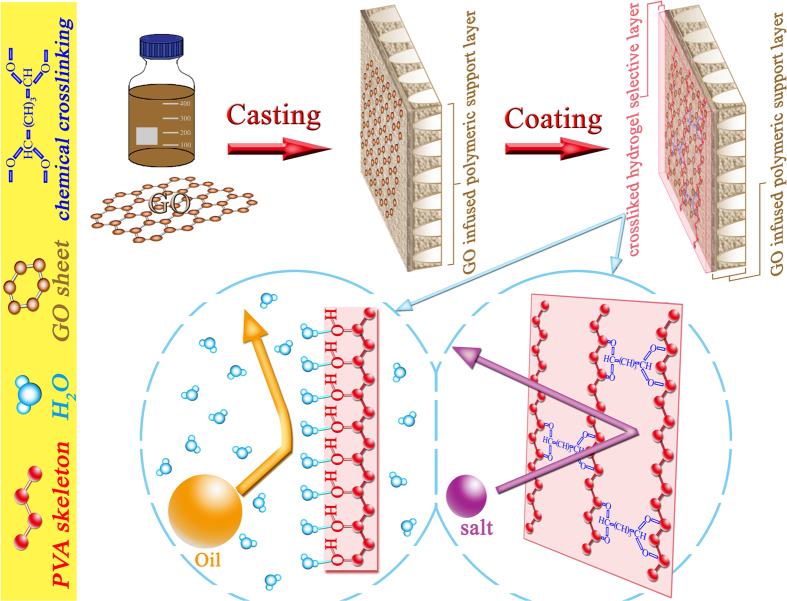
Illustration of the synthetic process (a–c) and work mechanisms (d,e) of Hydrogel/GO FO membrane. (**a**) Graphene oxide (GO) nanosheets are prepared and homogeneously dispersed in the polymer (PES) dope solution. (**b**) GO infused polymeric support layer is casted by phase inversion technique. (**c**) The hydrogel (PVA) is chemically crosslinked and coated on the surface of GO infused polymeric support layer. (**d**) The upmost surface of the hydrogel layer undergoes hydration in water to create an oil-repellent water barrier. (**e**) Simultaneously, the crosslinked structure of the hydrogel layer endows itself with the capability of rejecting salt ions efficiently. The molecular structures of the hydrated and crosslinked hydrogel layer are illustrated in (**d**) and (**e**), respectively. This figure was drawn by the author Mr. Detao Qin.

As illustrated in Fig. 1a–c, the synthesis of oil-repelling and salt-rejecting nanocomposite FO membrane (designated as “Hydrogel/GO FO membrane” in this study) consists of three major steps: (1) preparing GO nanosheets doped polymer solution, (2) casting GO infused polymeric support layer, (3) coating chemically-crosslinked hydrogel (PVA) selective layer (see molecular structures of individual chemicals in Figure S4). Figure 1c,d demonstrate the working mechanisms of this nanocomposite FO membrane.

**Figure 2:**
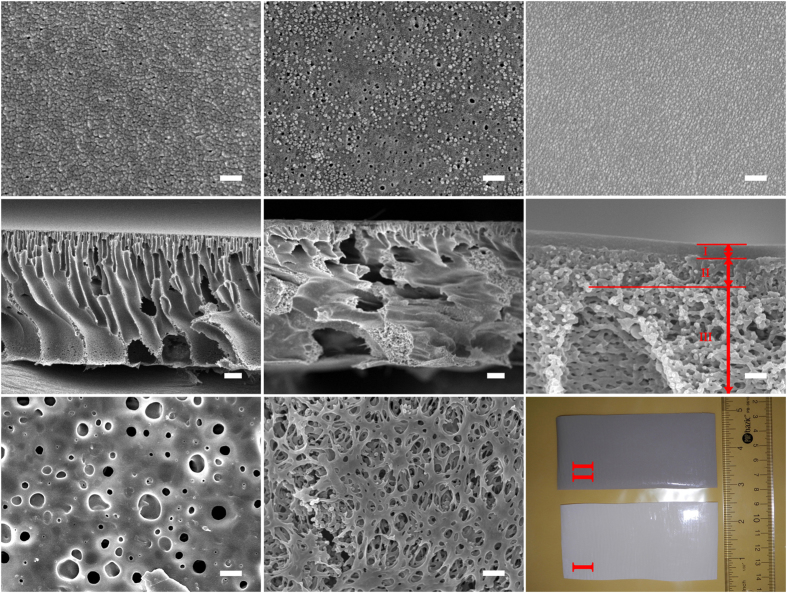
Investigation of as-synthesized membrane structures. (**a**) Top surface of pristine polymeric (PES) support layer (scale bar, 100 nm). (**b**) Top surface of GO infused polymeric support layer (scale bar, 100 nm). (**c**) Top surface of Hydrogel/GO FO membrane (scale bar, 100 nm). (**d**) Cross-sectional overview of pristine polymeric support layer (scale bar, 10 μm). (**e**) Cross-sectional overview of GO infused polymeric support layer (scale bar, 10 μm). (**f**) Enlarged cross-sectional image of Hydrogel/GO FO membrane (scale bar, 200 nm). Layer I is crosslinked hydrogel (PVA) selective layer; layer II is sponge-like sublayer of GO infused polymeric support layer; and layer III is macroporous sublayer of GO infused polymeric support layer. (**g**) Bottom surface of pristine polymeric support layer (scale bar, 1 μm). (**h**) Bottom surface of GO infused polymeric support layer (scale bar, 1 μm). (**i**) Optical photograph of pristine (I, white piece) and GO infused (II, dark grey piece) polymeric support layers.

An array of polymeric support layers were cast by phase inversion technique through differing GO content from 0.00 wt% to 0.30 wt% (see the characterization of GO nanosheet and its dope solution in Supplementary Discussions 4.1). Figure 2 demonstrates that the incorporation of GO nanosheets brings about significant changes in the physical structure of as-synthesized^49^ polymeric support layer. This is because the superhydrophilicity of GO nanosheets renders polymer/solvent/nonsolvent ternary system thermodynamically more instable^50^,^51^. As a result, Fig. 2a,b show that the pore size of support layer top surface is enlarged from 10 ± 1.6 nm to 20 ± 5.8 nm as GO weight fraction increased from 0.0% to 0.2%. Meanwhile, Fig. 2d,e show that the width of finger-like channels in support layer cross-section turns to be 2 ~ 3 times bigger, signifying improved support layer structure that is more favorable for water diffusion. And what is not pointed out by previous reports^52^,^53^ but was clearly found for the first time by us is that the spongy walls of finger-like channels become thicker in GO infused polymeric support layer. Moreover, Fig. 2g,h show that the pore size of support layer bottom surface is enlarged from 0.62 ± 0.18 μm to 1.35 ± 0.62 μm, implying the interconnectivity of pores also gets enhanced in support layer. In short, doping GO nanosheets shape as-synthesized support layer to be more porous.

**Figure 3:**
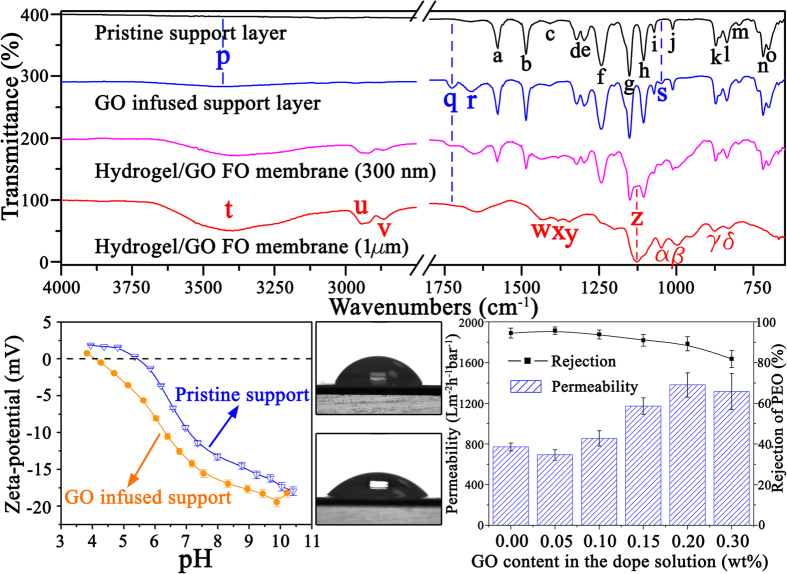
Investigation of surface chemistries and water fluxes of as-synthesized support layers. (**a**) ATR-FTIR spectra of pristine polymeric support layer, GO infused polymeric support layer, and Hydrogel/GO FO membranes (with hydrogel selective layer thickness of 300 nm and 1 μm, respectively).The details of IR band assignments are elaborated in Table S3. (**b**) Surface charges of pristine and GO infused polymeric support layers at different pH values. (**c**,**d**) Water contact angles of pristine and GO infused polymeric support layers, respectively. (**e)** Effect of GO content on water permeability and solute rejection of as-synthesized polymeric support layer.

The incorporation of GO nanosheets also generates significant impacts on the chemical properties of as-synthesized polymeric support layer. As shown in Fig. 3a, ATR-FTIR spectra confirm that the incorporation of GO nanosheets introduces hydroxyl (3433 cm^−1^, peak p), carboxyl (1726 cm^−1^, peak q) and epoxy groups (1050 cm^−1^, peak s) into polymeric support layer. Meanwhile, zeta-potential characterization results reveal that GO infused polymeric support layer carries more negative charges on top surface than the pristine one in a broad pH range (pH 3–11), with the electrostatic point decreased from pH 5.45 to pH 4.15 as GO weight fraction increased from 0.0% to 0.2% (Fig. 3b). This is because the embedded GO nanosheets equip the support layer surface with carboxylic acids as well as other oxygenic functional groups. And because these anchored oxygenic functional groups have better affinities for water molecules, water contact angle of support layer top surface is also reduced from 71 ± 3.8° to 50 ± 4.6° (Fig. 3c,d). The enlargement of surface pore size together with the enhancement of hydrophilicity explains the improvement in pure water permeability of polymeric support layer from 622 ± 41 L m^−2^ h^−1^ bar^−1^ to 1380 ± 119 L m^−2^ h^−1^ bar^−1^ as GO weight fraction raised from 0.0% to 0.2%. However, further increasing GO weight fraction from 0.2% to 0.3% does not show such obvious improvement in water permeability but compromised the selectivity of polymeric support layer (Fig. 3e). This implies that increasing GO content in the dope solution might incur the potential risk of forming defects on subsequently coated hydrogel selective layer. Therefore, GO weight fraction was fixed as 0.2% for the following experiments.

The hydrogel selective layer was further synthesized through coating glutaraldehyde (GA) crosslinked PVA on both pristine and GO infused polymeric support layers. PVA nanograins in 6 ~ 8 nm size were assembled into the orderly arrays, forming the compact, ultrasmooth (average roughness < 10 nm) and nm-defect-free selective layer (Fig. 2c). Cross-sectional SEM image indicates that this hydrogel selective layer is immobilized on the top of nanocomposite polymeric support layer at a uniform thickness of 110 ±  7 nm (Fig. 2f). This also demonstrates that as-synthesized Hydrogel/GO membrane possesses approximately ideal FO membrane structure^54^, which can be summarized as an ultrathin selective layer (layer I) sitting on hierarchically structured support layer that consists of a sponge-like skin sublayer (layer II) on top of a macroporous sublayer with finger-like channels (layer III). In addition, the characteristic band at 1132 cm^−1^ (peak z) on the ATR-FTIR spectra of Hydrogel/GO membrane (Fig. 3a), which refers to the stretching vibrations of C-O-C groups in the formed acetal bridges, confirms that the hydroxyl groups of PVA are successfully crosslinked by the aldehyde groups of GA (see Supplementary Discussions 4.2).

Table 1 summarizes the impacts on FO membrane intrinsic properties resulted from incorporating GO nanosheets, which can be elucidated in the following three perspectives. Firstly, embedding GO nanosheets (0.2% of the entire dope solution) into polymeric support layer leads to a marked improvement in the water permeability of FO membrane by 35% without compromising its salt rejection. Consequently, water flux of as-synthesized FO membrane is increased to 16.1 L m^−2^ h^−1^ (Draw solution is 0.5 M Na_2_SO_4_), which is 2.3 times higher than that of HTI FO membrane. This also means our Hydrogel/GO FO membrane is comparable to or better than the recently reported FO membranes with nanofiller embedded support layer in terms of *J*_***V***_ performance^55^,^56^,^57^,^58^. Secondly, the reverse salt leakages (*J*_*S*_) of as-synthesized FO membranes are comparable to or even lower than that of HTI FO membrane, leading to an ultralow *J*_*S*_*/J*_***V***_ (0.06 ~ 0.08 g/L) which is only 1/4 ~ 1/3 as that of HTI membrane. This means as-synthesized FO membranes outperform HTI FO membrane in the operation cost of draw solute replenishment. Thirdly and most importantly, FO membrane structural parameter (*S* value) is reduced by 20% from 244 μm (“Hydrogel” membrane) to 197 μm (“Hydrogel/GO membrane”), substantiating that embedding GO nanosheets into support layer functions as an effective technique to mitigate ICP. In detail, two parts contribute to the smaller *S* value. One contributor is the increase of membrane porosity (*ε*), implying the enhancement of support layer wettability. The other contributor that plays the major role is the significant decrease of membrane tortuosity (*τ*) from 2.5 to 2.0, which is consistent with the improvement in pore interconnectivity of support layer as aforementioned.

### Evaluation of FO membrane fouling-resistance

**Figure 4:**
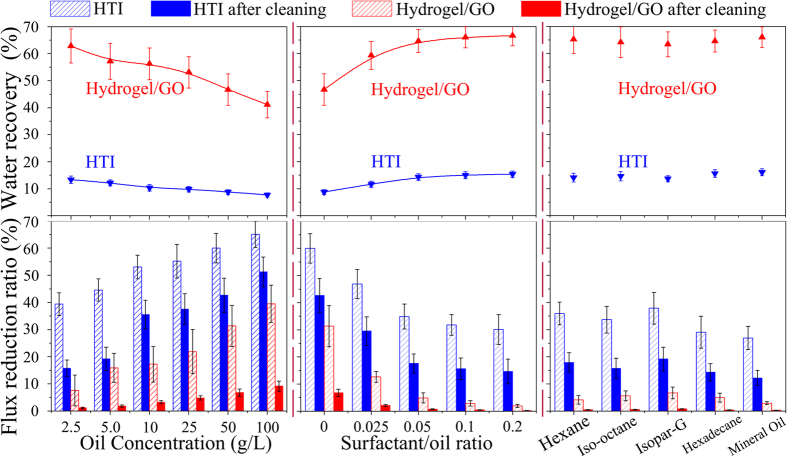
Water recoveries (upper panel) and flux reduction ratios (lower panel) of Hydrogel/GO and HTI FO membranes. (**a**) The effect of oil concentrations (The surfactant concentration is zero). (**b**) The effect of surfactant/oil ratios (The oil concentration is 50 g/L). (**c**) The effect of different kinds of oils (The oil concentration is 25 g/L and the surfactant/oil ratio is 0.05). The experiment condition is using 1.5 M Na_2_SO_4_ as draw solution.

It’s believed that FO process provides a unique scenario to investigate the susceptibility of salt-rejecting membrane to certain foulants, because fouling associated with hydraulic pressure is minimized or negligible. In order to exclude the interferences of salinity and surfactant, herein feed solutions were prepared by sonicating oil (vegetable oil) in DI water at different oil concentrations. Figure 4a shows that as oil concentration increased, membrane fouling is aggravated and hence water recovery at the given operation time is reduced; nevertheless, under all circumstances compared with commercial HTI FO membrane, Hydrogel/GO FO membrane achieves better fouling-mitigation in terms of lower flux reduction ratio (FRR) together with much higher water recovery. In detail, Hydrogel/GO FO membrane is >50% lower than HTI FO membrane in *FRR*_*f*_ (FRR in “oil-fouling stage”) under 2.5 ~ 50 g/L oil concentrations. Even fed with ultrahigh oil concentration like 100 g/L, the *FRR*_*f*_ of Hydrogel/GO membrane is 39.5%, still 40% lower than the *FRR*_*f*_ of HTI membrane that is 65.1%; correspondingly, Hydrogel/GO membrane achieves 41% water recovery, surpassing HTI membrane that achieves only 7.7% water recovery. Furthermore, most of the *J*_*V*_ losses can be recovered for Hydrogel/GO FO membrane through *in-situ* washing by DI water, leading to its *FRR*_*c*_ (FRR in “post-cleaning stage”) ranged from 1.1% to 9.2%. On the contrary, the DI water cleaning effect for HTI FO membrane is poor, resulting in much higher *FRR*_*c*_ ranged from 15.8% to 51.3%.

For the purpose of studying membrane fouling under surfactant-stabilized emulsions, Triton X-100 was added into 50 g/L oil-in-water emulsion in different surfactant/oil ratios. Figure 4b shows that as surfactant/oil ratio increased, fouling is mitigated and hence water recovery is restored; more importantly, the superiorities in fouling-resistance and water recovery of Hydrogel/GO membrane are strengthened over HTI membrane. In detail, the *FRR*_*f*_ of HTI membrane is reduced from 60.1% to 34.9% as surfactant/oil ratio increased from 0.00 to 0.05. Nevertheless, further increasing surfactant/oil ratio to 0.20 is ineffective to continue such remarkable improvement in fouling-mitigation for HTI membrane, with its *FRR*_*f*_ and *FRR*_*c*_ stabilized around 31% and 15%, respectively. On the contrary, the *FRR*_*f*_ of Hydrogel/GO FO membrane is reduced from 31.4% to 4.9% through increasing surfactant/oil ratio from 0.00 to 0.05. And above 0.05 surfactant/oil ratio, fouling of Hydrogel/GO FO membrane is negligible, for its *FRR*_*f*_ reduced to <3.0% accompanied by approximately zero *FRR*_*c*_. Consequently, as surfactant/oil ratio ≥0.05%, Hydrogel/GO FO membrane achieves ~65% water recovery, surpassing HTI FO membrane that obtains only ~15% water recovery.

To substantiate the universality of Hydrogel/GO membrane’s excellent antifouling capability, petroleum oils of different carbon numbers were selected to prepare emulsions with oil concentration and surfactant/oil ratio fixed as 25 g/L and 0.05, respectively. Noteworthily, there is no obvious correlation between carbon number of oil molecule and fouling tendency as expected. Figure 4c shows that Hydrogel/GO FO membrane exhibits ultralow fouling extents with its *FRR*_*f*_ ranged from 2.9% to 6.7% and *FRR*_*c*_ below 1.0%. On the contrary, HTI FO membrane suffers much severer flux losses with its *FRR*_*f*_ ranged from 26.9% to 37.9% and *FRR*_*c*_ ranged from 12.2% to 19.2%, respectively. Consequently, in terms of water recovery HTI membrane (~15%) is outclassed by Hydrogel/GO membrane (~64%) when investigated with emulsions prepared from different petroleum oils.

**Figure 5:**
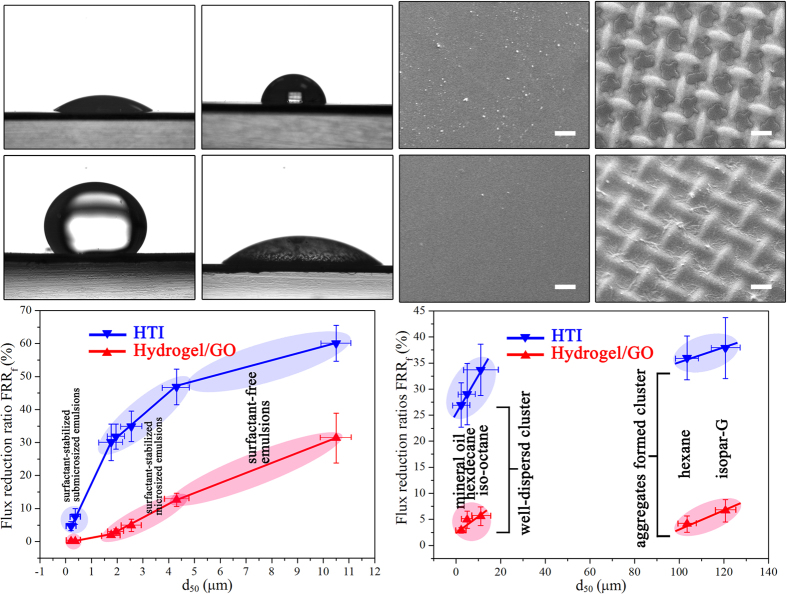
Different responses to oil-fouling between Hydrogel/GO FO membrane and HTI FO membranes. (**a**,**b**) Water contact angle and underwater oil contact angle of Hydrogel/GO FO membrane, respectively. (**c**,**d**) Water contact angle and underwater oil contact angle of HTI FO membrane, respectively. (**e**,**f**) SEM images of Hydrogel/GO FO membrane surface after oil-fouling test and *in-situ* cleaning, respectively (scale bar, 100 μm; the oil concentration is 25 g/L and the surfactant/oil ratio is zero). (**g**,**h**) SEM images of HTI FO membrane surface after oil-fouling test and *in-situ* cleaning, respectively (scale bar, 100 μm; the oil concentration is 25 g/L and the surfactant/oil ratio is zero). (**i**) Water flux reduction ratio as a function of average oil droplet size (*d*_*50*_) for the same kind of oil (The oil is vegetable oil). (**j)** Water flux reduction ratio as a function of average oil droplet size (*d*_*50*_) under different petroleum oils (The oil concentration is 25 g/L and the surfactant/oil ratio is 0.05).

The exceptional anti-fouling capability of Hydrogel/GO FO membrane under various oil/water emulsions can be mainly attributed to its superior surface wettability. In detail, Hydrogel/GO membrane surface is highly hydrophilic and underwater oleophobic, with 30 ± 3.6° water contact angle (Fig. 5a) and 141 ± 4.5° underwater oil contact angle (Fig. 5b). The synthesized hydrogel selective layer undergoes hydration in water and thus endows itself with strong oil-repellency that leads to the high fouling-resistance. On the contrary, HTI membrane surface is weak in hydrophilicity and strong in underwater oleophilicity, with 87 ± 4.1° water contact angle (Fig. 5c) and 35 ± 5.7° underwater oil contact angle (Fig. 5d). The strong affinity of HTI membrane surface for oil induces the oil adsorption from feed emulsions. Consequently, Hydrogel/GO membrane is distinct from HTI membrane in surface response to oil-fouling. In detail, only a small amount of oil aggregates is able to settle on Hydrogel/GO membrane surface during “oil-fouling stage” (Fig. 5e). The loose attachment between hydrogel selective layer and oily foulant renders most of these oil aggregates washed away by *in-situ* DI water cleaning (Fig. 5f). In contrast, the feed oil covers almost all the surface area of HTI membrane and agglomerates into ~10 μm thick cake layer at the concave parts (Fig. 5g), wherein the hydraulic flow is lacking in shear force. The strong adhesion of oily foulant to HTI membrane surface makes i*n-situ* washing only able to extrude part of this cake layer and thus leave an oil film stuck on membrane surface (Fig. 5h), explaining the poor flux restoration of HTI membrane after cleaning.

To further understand other factors also influencing membrane response to oil-fouling, dynamic light scattering (DLS) and optical microscope were used to examine oil droplet size distributions. Figure S10 elucidates that for the same kind of oil, increasing surfactant/oil ratio can narrow oil droplet distribution towards smaller size; nevertheless, such effectiveness is limited within the scope of micrometer sized emulsions. And to obtain submicrometer sized emulsions, oil concentration must be controlled under a reasonable level (*e.g.* <5 g/L in this study). Figure S11 indicates that among different petroleum oils, the oil droplets of iso-octane, hexadecane and mineral oil remain detached without aggregation in emulsions, while the oil droplets of hexane and isopar-G can cohere into macroaggregates as large as 100 ~ 500 μm. Based on these results, mathematical fittings between oil droplet size distributions (it terms of mass median diameter *d*_*50*_) and fouling extents are analyzed for both Hydrogel/GO and HTI FO membranes. Figure 5i demonstrates that for the same kind of oil, the bigger oil droplet in size, the heavier FO fouling would be, possibly because oil droplet in bigger size has larger surface area and hence stronger attraction force with membrane surface. More importantly, underwater oleophilic surface *i.e.* HTI membrane is more susceptible to oil droplet in larger size. This is indicated by the fact that the *FRR*_*f*_-*d*_*50*_ curve slope of HTI membrane turns to be much steeper compared with that of Hydrogel/GO membrane when *d*_*50*_ exceeds 0.5 μm. Meanwhile, Fig. 5j demonstrates that for different petroleum oils the data points of *FRR*_*f*_ can be grouped into two clusters based on the dispersibility of oil. There is no obvious correlation between *d*_*50*_ and *FRR*_*f*_ throughout data clusters for either Hydrogel/GO membrane or HTI membrane. This result implies that factors other than physical size of oil droplet, e.g. chemical affinity between oil and surface as discussed previously, could also influence FO membrane fouling. Additional discussion on Fig. 5i–j is provided in SI (Supplementary Discussions 4.3).

### Simulated shale gas wastewater treatment

**Figure 6:**
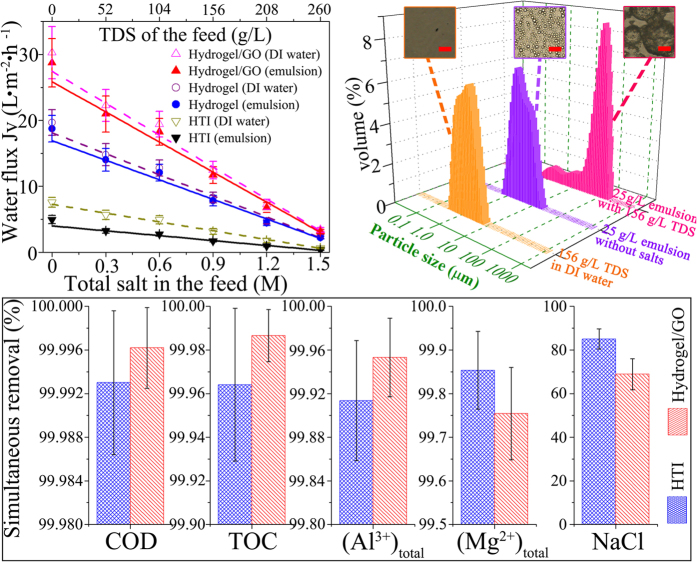
The study of simultaneously deoilling and desalting shale gas wastewaters. (**a**) Water fluxes of FO membranes as a function of salinity in feed solution (Draw solution is 1.5 M Na_2_SO_4_. Dotted lines represent studies without oil, while solid lines represent studies with surfactant-stabilized hexadecane-in-water emulsions; “Hydrogel” represents synthesized FO membrane with pristine polymeric support layer, while “Hydrogel/GO” represents synthesized FO membrane with GO infused polymeric support layer.). (**b**) Salt/oil particle size distributions in different feed solutions. The inset figures are optical microscopic images of different feed solutions, scale bar, 50 μm. (**c**) Simultaneous removals of oil and salts from shale gas wastewater by FO membranes (Feed solution is hexadecane-in-water emulsion with 25 g/L oil concentration, 0.05 surfactant/oil ratio and 156 g/L TDS. Draw solution is 1.5 M Na_2_SO_4_).

In order to simulate shale gas wastewater, inorganic salts including NaCl, MgSO_4_ and Al_2_(SO_4_)_3_ were added into hexadecane-in-water emulsions in the range of 0 ~ 260 g/L total dissolved salts (TDS) with oil concentration and surfactant/oil ratio fixed as 25 g/L and 0.05, respectively. Generally, Hydrogel/GO membrane can achieve more than 3 times higher FO water flux compared with HTI membrane when investigated with simulated shale gas wastewater. In detail, the solid lines on Fig. 6a demonstrates that as TDS of hexadecane-in-water emulsions increased from 0 g/L to 260 g/L, FO water fluxes are lowered down almost linearly due to the diminution of osmotic driving force, with *J*_*V*_ reduced from 28.7 ± 3.7 L m^−2^ h^−1^ to 3.2 ± 0.3 L m^−2^ h^−1^ for Hydrogel/GO membrane, and reduced from 5.0 ± 0.6 to 0.3 ± 0.1 L m^−2^ h^−1^ for HTI membrane, respectively (Draw solution is 1.5 M Na_2_SO_4_). More importantly, at any particular TDS compared with HTI FO membrane, the synthesized FO membranes are smaller in the absolute value of *J*_*V*_ loss resulted from oil-fouling, which can be represented by the vertical distance between dash line and solid line of each membrane on Fig. 6a. This means that Hydrogel/GO membrane maintains its highly antifouling advantage over HTI membrane under salinity-existed oil/water emulsions.

To further understand how the existence of salts in emulsion complicates membrane fouling, *J*_*V*_-time functions under both salinity-free emulsions (including surfactant-free emulsion as well as surfactant-stabilized emulsion) and simulated shale gas wastewater were systematically investigated and presented in Figure S12. Noteworthily, for HTI FO membrane the *FRR*_*f*_ of simulated shale gas wastewater (~45.9%) is evidently higher than that of salinity-free emulsion (~34.6%) at the same oil concentration (25 g/L) and surfactant/oil ratio (0.05). This can be attributed to two reasons. One reason is that salinity-induced agglomeration of oil droplets aggravates the fouling extent for underwater oleophilic surface. In reality, shale gas wastewater usually contains high concentrations of scale-forming constituents^4^, which can develop into colloids or precipitates and further trigger the aggregation of oil droplets. Correspondingly, both DLS and optical microscopy results confirm that the average oil droplet size (*d*_*50*_) is increased from 3.0 μm to 67.3 μm as TDS of emulsion increased from 0 g/L to 156 g/L (Fig. 6b). Consequently, HTI FO membrane suffers even severer loss of permeability at higher feed salinity because it is more susceptible to oil droplet in larger size as mentioned previously. The other reason could be cake enhanced osmotic pressure (CEOP)^17^ arisen from the synergistic effect between salts and oil-fouling in feed solution. In detail, on HTI membrane surface micrometer sized oil droplets can agglomerate by adsorption and further grow into a cake layer as thick as 10 μm, within which the diffusion of salt ions are significantly hindered. As a result, in the feed side TDS is accumulated to a much higher level at membrane surface than the bulk of solution, which means the effective osmotic gradient across HTI membrane is dramatically reduced. On the contrary, for Hydrogel/GO FO membrane the *FRR*_*f*_ of shale gas wastewater (7.9%) is slightly higher than that of salinity-free emulsion (5.2%) at the same oil concentration and surfactant/oil ratio, indicating that the superior fouling-resistance of as-synthesized hydrogel selective layer is robust under hypersaline oil/water emulsions.

**Figure 7:**
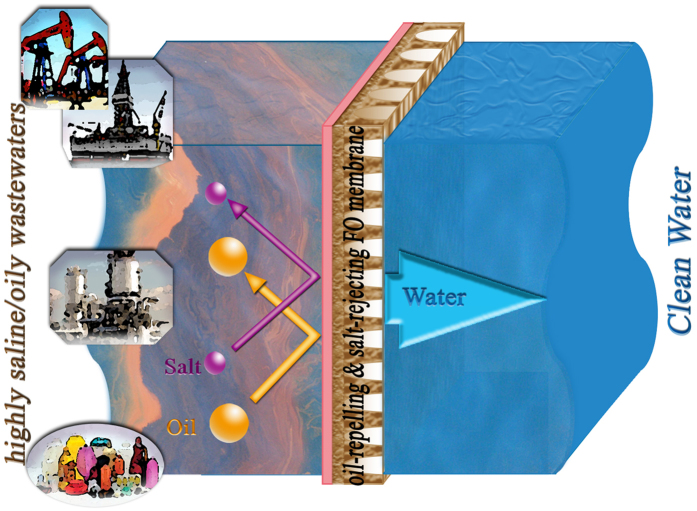
Schematic diagram of simultaneous oil/water separation and desalination by Hydrogel/GO FO membrane. This figure (including the inserted subfigures of oil exploitation, oil refinery and petroleum products) was drawn by the author Mr. Detao Qin.

Furthermore, clean water can be obtained as a result of simultaneously deoilling and desalting shale gas wastewater by Hydrogel/GO FO membrane (Figure S13). Table S6 presents water quality analysis results of both feed and draw solutions at the end of “oil-fouling stage”, based on which simultaneous removals of oil and ions by FO process are calculated according to equation (6) and shown in Fig. 6c. Both Hydrogel/GO and HTI FO membranes can reach >99.99% removal of COD and >99.9% removal of TOC, indicating all organic pollutants in simulated shale gas wastewater are rejected. And more importantly, Hydrogel/GO membrane outperforms HTI membrane in oil removal, which is mainly attributed to the strong underwater oleophobicity of as-synthesized hydrogel selective layer. Meanwhile, Hydrogel/GO FO membrane also demonstrates ~99.95% removal of Al^3+^ and ~99.75% removal of Mg^2+^, which is slightly higher than or comparable to that of HTI FO membrane, respectively. In addition, Hydrogel/GO FO membrane can achieve ~69% removal of Cl^−^. Though its removal of monovalent ion is lower than that of HTI membrane (~85% removal of Cl^−^), this result indicates that chemically-crosslinked hydrogel (PVA) layer is able to reach NF to RO selectivity. In short, as illustrated in Fig. 7, Hydrogel/GO FO membrane is capable of simultaneously deoilling and desalting shale gas wastewater with its hydrogel selective layer rejecting all organic pollutants and most inorganic ions while transporting water molecules at high flux and low fouling tendency.

## Discussion

The major obstacle in implementing membrane technology to treat shale gas wastewater is the lack of a membrane that can simultaneously possess two functions: oil-repellency and salt-rejection. Conventional salt-rejecting membranes (such as polyamide RO membrane) are hydrophobic (oleophilic), which results in severe membrane fouling during oil/water separation. This study reveals that certain crosslinked hydrogels can serve as the bifunctional selective layer that possesses both oil-repelling and salt-rejecting properties. On one hand, the upmost surface of the hydrogel selective layer undergoes hydration in water and bonds water molecules tightly to create an ultrathin water barrier. And this hydrated hydrogel can repel oil adhesion owing to the dehydration entropic effect^59^,^60^, thus leading to the low fouling tendency. More interestingly, the synthesized hydrogel FO membranes exhibit robust resistance to salinity-induced fouling aggravation under hypersaline oil/water emulsions. On the other hand, the hydrogel polymer chains can be bridged through covalent-bonded chemical crosslinking, and thus the three-dimensional (3D) macromolecular network can be built. As a result, the synthesized FO membranes also possess ultrahigh rejections of multivalent inorganic ions as well as emulsified oils. Herein, the infused GO nanosheet plays a crucial role to improve FO membrane structure (reducing *S* value) by reducing the tortuosity as well as increasing the porosity of the support layer, and consequently lead to constantly high water flux for this new nanocomposite FO membrane. And the high water flux is an important element to make this new FO membrane economically feasible for practical treatment of shale gas wastewater. In addition, as-synthesized nanocomposite FO membrane achieves ~45% water recovery in treating simulated shale gas wastewater before reaching osmotic pressure balance (Draw solution is 1.5 M Na2SO4. Feed solution is 25 g/L hexadecane-in-water emulsion with 0.05 surfactant/oil ratio and 156 g/L TDS), showing the promise for practical application.

In summary, it is the first time to report a new nanocomposite FO membrane that accomplishes simultaneous oil/water separation and desalination for shale gas wastewater treatment. This nanocomposite FO membrane consists of an oil-repelling and salt-rejecting hydrogel selective layer on top of a GO infused polymeric support layer. The infused GO in support layer (0.2% of entire dope solution) is able to reduce structural parameter of as-synthesized FO membrane as much as 20%. And the hydrogel selective layer demonstrates strong underwater oleophobicity, which leads to superior antifouling property under hypersaline oil/water emulsions. Compared with commercial FO membrane, this new FO membrane can simultaneously deoil and desalt simulated shale gas wastewater with more than three times higher water flux, higher removal efficiencies for oil and salts (>99.9% for oil and >99.7% for multivalent ions), and significantly lower membrane fouling tendency.

## Additional Information

**How to cite this article**: Qin, D. *et al.* A new nanocomposite forward osmosis membrane custom-designed for treating shale gas wastewater. *Sci. Rep.* **5**, 14530; doi: 10.1038/srep14530 (2015).

## Supplementary Material

Supplementary Information

## Figures and Tables

**Table 1:** Intrinsic properties of as-synthesized and commercial HTI FO membranes.

Membrane	Water Permeability (Lm^−2^h^−1^bar^−1^)	Rejection of Na_2_SO_4_(%)	Water flux in FO mode (Lm^−2^h^−1^)	Reverse salt flux in FO mode (gm^−2^h^−1^)	Structural Parameter (μm)	Thickness (μm)	Porosity (%)	Tortuosity
HTI	0.39 ± 0.06	97.0 ± 1.0	4.92 ± 0.64	1.16 ± 0.28	453 ± 45	52 ± 11	41.0 ± 2.7	3.62 ± 0.27
Hydrogel	1.13 ± 0.10	94.0 ± 1.3	11.49 ± 1.14	0.70 ± 0.25	244 ± 27	80 ± 7	80.5 ± 1.6	2.54 ± 0.18
Hydrogel/GO	1.52 ± 0.12	92.2 ± 1.5	16.05 ± 1.40	1.27 ± 0.44	197 ± 21	84 ± 8	85.2 ± 1.3	2.02 ± 0.11

Note: “Hydrogel” represents synthesized FO membrane with pristine polymeric support layer, while “Hydrogel/GO” represents synthesized FO membrane with GO infused polymeric support layer. For RO test, the feed solution is 10 mM Na_2_SO_4_ under 5  bar; for FO test, the feed solution is DI water and the draw solution is 0.5 M Na_2_SO_4_.
