# Supplementary material for: A new nanocomposite forward osmosis membrane custom-designed for treating shale gas wastewater
Source: Sci Rep. 2015 Sep 29;5:14530. doi: 10.1038/srep14530 (PMC4586890; doi:10.1038/srep14530)
Supplement: Supplementary Information [file srep14530-s1.pdf]

Supplementary Information (SI)

A new nanocomposite forward osmosis membrane custom-designed for treating shale gas wastewater

Detao Qin[1], Zhaoyang Liu[2,*], Darren Delai Sun[3,*], Xiaoxiao Song[3], Hongwei Bai[4]

[1]*Energy Research Institute @ NTU, Interdisciplinary Graduate School, Nanyang Technological University, 639798, Singapore*

[2]*Qatar Environment and Energy Research Institute, Qatar Foundation, PO Box 5825, Doha, Qatar. E-mail: zhliu@qf.org.qa; Fax: +974 4454 0547; Tel: +974 4454 5621*

[3]*School of Civil and Environmental Engineering, Nanyang Technological University, 639798, Singapore. E-mail: ddsun@ntu.edu.sg; Fax: +65 6791 0676; Tel: +65 6790 6273*

[4]*Energy Research Institute @ NTU, Nanyang Technological University, 639798, Singapore*

This PDF file includes:

1. Supplementary Introduction (page S2-S3)
2. Supplementary Experiment Details (page S4-S11)
3. Supplementary Figures and Tables (page S12-S25)
4. Supplementary Discussions (page S26-S32)
5. Reference of Supplementary Information (page S33-S34)

1. Supplementary Introduction

The concept of internal concentration polarization (ICP)

Internal concentration polarization (ICP) is a very unique phenomenon that takes place in osmotically-driven membrane processes. In detail, ICP refers to the mechanism that the support layer of FO membrane functions as an unstirrable barrier to the diffusion of draw solutes thus resulting in a significantly lower osmotic gradient across membrane selective layer (effective osmotic driving force, $\Delta\pi_{eff,}$ as shown in Figure S1) than the osmotic difference between the bulks of feed and draw solutions (apparent osmotic driving force, $\Delta\pi_{bulk}$, as shown in Figure S1). Specifically, in FO mode (selective layer facing feed solution, which is employed in this study), as water permeates through membrane selective layer, the draw solution within the support layer is being diluted. As a result, the effective osmotic driving force across membrane selective layer is diminished because the osmotic pressure at the interface between selective layer and support layer ($\pi_{D,eff}$, as shown in Figure S1) is significantly lower than the bulk of draw solution ($\pi_{D,b}$, as shown in Figure S1).

And the governing equation for permeate flux in FO mode considering ICP effect is developed by published peer studies[1] and adapted here.

$$J_v = A\,\Delta\pi_{eff} = A\left(\pi_{D,eff} - \pi_{F,m}\right) = A(\pi_{D,m}\exp(-J_v K) - \pi_{F,m}) \qquad \text{(S1)}$$

where A is the intrinsic water permeability of FO membrane, $\Delta\pi_{eff}$ is the effective osmotic driving force across membrane selective layer, $\pi_{D,eff}$ is the osmotic pressure of draw solution at the interface between selective layer and support layer, $\pi_{F,m}$ is the

osmotic pressure of feed solution at membrane surface (selective layer top surface), $\pi_{D,m}$ is the osmotic pressure of draw solution at membrane surface (support layer bottom surface), J_V is FO water flux, K is solute resistivity, and $exp(-J_vK)$ is termed as the dilutive ICP modulus, which is used to quantitatively analyze the adverse effect of ICP in FO mode.

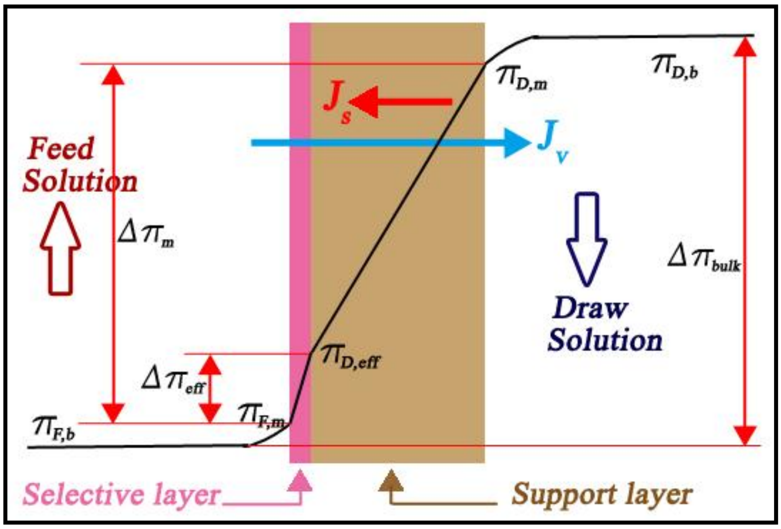

Figure S1. ICP across a composite membrane in FO mode (adapted from reference[2]).

It's evident that ICP effect and FO membrane structure parameter (S value, equaling to $K \times D$ where D is the diffusion coefficient of draw solute, see more details in Methods section of main text) are inextricably linked: the higher S value, the higher K value, the smaller ICP modulus, the server ICP effect. More importantly, unlike external concentration polarization (ECP), ICP cannot be mitigated through increasing crossflow velocity or turbulence on membrane surface. In other words, ICP is a more stubborn issue to FO process that is addressed mainly through improving FO membrane structure (reducing S value in terms of making the structure of support layer to be more porous, less tortuous as well as less thick).

2. Supplementary Experimental Details

2.1. Synthesis of graphene oxide (GO)

A modified Hummer's method was adopted to prepare GO nanosheets. In detail, 14 ml 98% sulfuric acid was added into the mixture of 0.5 g graphite flakes (SP 1 Bay Carbon) and 2.0 g $NaNO_3$. The mixture was stirred for 30 min while being cooled to 0 °C in an ice-water bath. 3.0 g $KMnO_4$ was added into the mixture slowly prior to stirring the mixture at 0 °C for another 2 hours. Then external heating was introduced to warm the reaction to 35 °C for 30 min. After that 40 ml deionized (DI) water was added into the mixture. The reaction temperature was further increased to 100 °C for 15 min and then the mixture was cooled down to room temperature before diluted with 70 ml DI water. The color of dispersion was changed immediately from dark red to bright yellow as 10 ml 35% H_2O_2 added. The resultant dispersion was centrifuged and resuspended in 10% HCl for three times to remove impurities, followed by washed with DI water several times to adjust pH value. After that, the precipitates were freeze-dried for at least 2 days to obtain graphite oxide. Finally, graphene oxide (GO) nanosheets were produced by the exfoliation of as-synthesized graphite oxide.

2.2. Determination of FO water flux (J_V) and reverse salt flux (J_S).

A custom-built FO system equipped with cross-flow cell was used to determine membrane performance (Figure S2). Both feed and draw solutions were circulated by gear pumps (Cole-Parmer) at flow velocity of 21.4 cm s^{-1} under 22 ± 1 °C with spacers (SEPA CF spacer, 17 mil) placed on both sides in the cell to increase

turbulence; and under this crossflow condition external concentration polarization (ECP) effect was rendered negligible. Water flux (J_V) and reverse salt flux (J_S) were recorded online according to the following equations (S2-S3):

$$J_v = \frac{\Delta V_{DS}}{A_m \times \Delta t} = \frac{\Delta m_{DS}}{\rho \times A_m \times \Delta t} \quad \text{(S2)}$$

$$J_S = \frac{\Delta(c \times V_{FS}) \times MW}{A_m \times \Delta t} \quad \text{(S3)}$$

where Δt is the time interval (2 min), A_m is the effective membrane area (23.8 cm^2), V_{DS} is the volume of draw solution, m_{DS} is the mass of draw solution, ρ is the density of water; V_{FS} is the volume of feed solution, c_{FS} is the molar concentration of draw solute in the feed solution (converted from calibrated conductivity, COND610, Eutech) and MW is the molecular weight of draw solute.

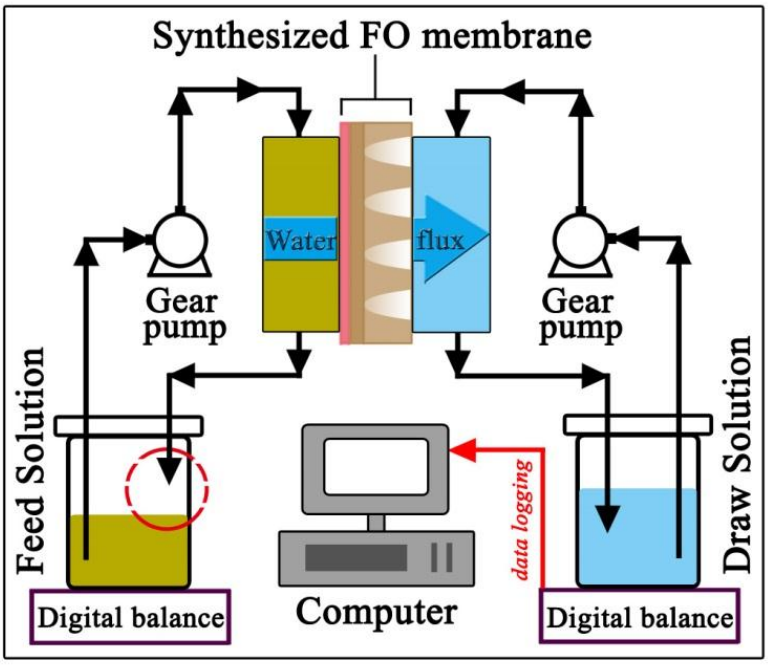

Figure S2. Schematic diagram of the custom-built FO setup. Note that in the feed tank the returning tubing tip of concentrate was placed 3 cm higher than water level (as marked by the dash-line circle).

2.3 The rationales for selecting different oils

The rationales for choosing these different kinds of oil to represent petroleum products are elaborated as follows. Hexane (*n*-hexane, C_6H_{14}) is the alkane that is in stable liquid form at room temperature (boiling point ~69 °C) with the smallest carbon number in molecule. Although pentane (C_5H_{12}) is also in liquid form, it is not chosen in this study because its boiling point is as low as 36 °C. 2,2,4-trimethylpentane (iso-octane, $(CH_3)_3CCH_2CH(CH_3)_2$) is an important component of gasoline. This particular isomer of octane is set as the standard 100 point on the 'octane number' rating scale. And it can be used in large proportions to increase the knock resistance of gasoline[3]. Isopar-G is a typical isoparaffin liquid thus utilized to represent branched aliphatic hydrocarbons. It is produced through distilling crude oil at temperature 161~173 °C and it has 10~11 carbon atoms in one molecule[4]. n-Hexadecane (cetane, $C_{16}H_{34}$) is an important component of diesel fuel. This particular alkane hydrocarbon ignites very easily under compression. So it is assigned as the standard 100 point on the 'cetane number' rating scale, which is used to evaluate the detonation of diesel fuel[3]. Mineral oil is a mixture of hydrocarbons with 15~40 carbon atoms in one molecule, which is produced as the byproduct of petroleum distillation. The mineral oil used here is a commercially available pump lubricating oil produced from Vacuubrand, Wertheim Germany.

In addition, the composition of vegetable oil used in this study is elaborated in Table S1.

Table S1. The ingredients of vegetable oil used in this study. (Brand name: "Sunflower & Olive Oil"; purchased from local supermarket "Giant" at Singapore; this table is quoted directly from the product label.)

	Average quantity per serving (15 ml)	Average quantity per 100 ml
Energy	511 kJ<br>122 kcal	3416 kJ<br>816 kcal
Protein	0.0 g	0.0 g
Fat, total	13.6 g	90 g
——saturated fat	1.6 g	10.7 g
——trans fat	0.1 g	0.6 g
Cholesterol	0 mg	0 mg
Carbohydrate	0.1 g	0.7 g
Sugars, total	0.0 g	0.0 g
Dietary fiber	0.0 g	0.0 g
Sodium	0 mg	0 mg
Calcium	0 mg	0.1 mg

2.4. An important notice on placing the returning tubing tip of concentrate above water level to eliminate oil/water stratification during FO testing process

In order to ensure the feed solution was kept as the homogenous emulsion form during FO testing period, the returning tubing tip of concentrate in feed tank was placed 3 cm higher than water level. The reason behind this setting is if the returning tubing tip of concentrate is immersed in feed solution, the stratification of oil/water mixture would take place, and consequently, a concentrated oily layer would form on the top of water. This phenomenon is obvious especially when investigating surfactant-free emulsions or simulated shale gas wastewater. This means the oil concentration fed to membrane is being gradually reduced during FO operation period. To overcome this problem, the returning tubing tip of concentrate was purposely placed above water level (as marked in Figure S3) in order to keep generating strong

hydraulic mixing of feed solution especially in the vertical direction (Flow rate is 1.0 L/min, corresponding to flow velocity of 21.4 cm/s.). As a result, the feed solution was kept being emulsified because the strong hydraulic agitation is able to continue breaking oil aggregates into smaller ones and force them being mixed in the bulk of feed solution. Therefore, this setting of tubing serves as an uncomplex but very effective method to eliminate any stratification of oil/water mixture during FO testing period and thus ensure the membrane has confronted the oil concentration truly as high as designated.

Figure S3 gives an example when 100 g/L surfactant-free oil-in-water emulsion is used as feed solution. Figure S3(a-b) shows that even under ultrahigh oil concentration like 100 g/L (Surfactant concentration is zero.), the feed solution can be maintained as a homogenous milky emulsion without any oil/water stratification. Noteworthily, as marked by the red circle on Figure S3b, the returning tubing tip of concentrate in feed tank is placed 3 cm above water level. Figure S3c shows that oil droplets of feed solution are ranged from 5 to 80 μm in size under 100 g/L oil concentration, confirming that feed solution exists in the form of homogenous emulsion. During FO testing process, the oil/water mixture was periodically sampled from the returning tubing tip to measure the oil concentration. The red symbols on SI Figure S3d indicate that as water recovery increased along with operation time, the oil concentration being fed to membrane (in terms of g oil/L water, measured under the setting of placing returning tubing tip above water level) is also increased. This

increase of oil concentration is because water is recovered through permeating FO membrane while oil is retained in feed emulsion. And more importantly, the measured oil concentration is found to be in consistent with the theoretical value of oil concentration calculated based upon equation S4 (The theoretical value is indicated by the black line on Figure S3d.). In contrast, the blue symbols on Figure S3d indicate that the oil concentration being fed to membrane is gradually decreased when placing the returning tubing tip of concentrate below water level in feed tank, because under this setting a concentrated oily layer would form on top of water.

$$Theoretical\ oil\ conc.\ = Starting\ oil\ conc. \times \frac{1}{1-water\ recovery} \qquad \text{(S4)}$$

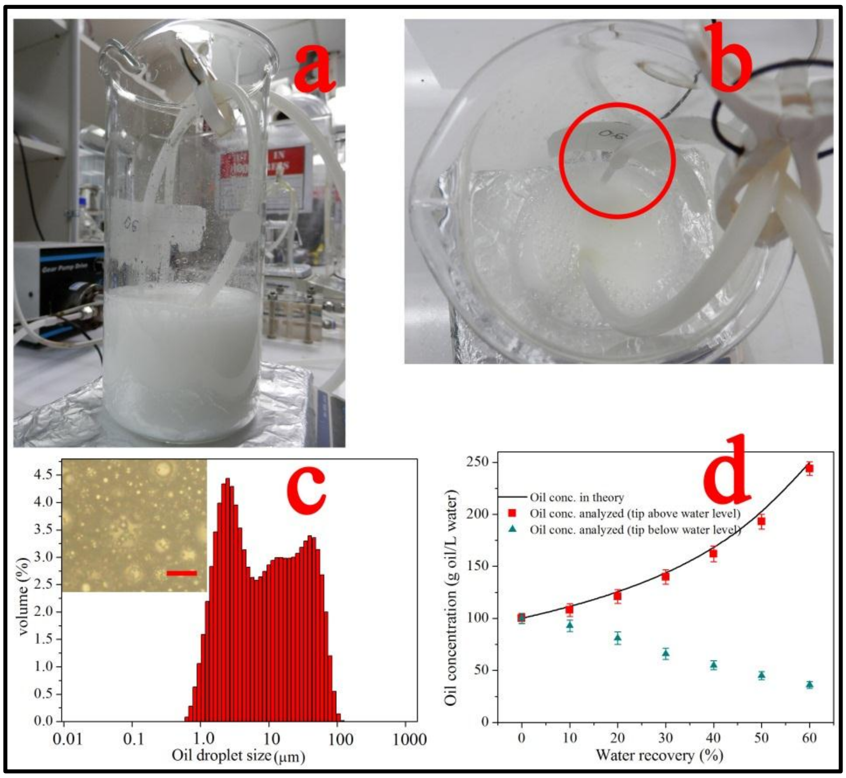

Figure S3. Placing the returning tubing tip of concentrate above water level in feed tank to eliminate oil/water stratification. Surfactant concentration is zero. (a, b) Optical photos of 100 g/L oil-in-water emulsion. (c) Optical microscopy image (inset, the scale bar is 50 μm) and dynamic light scattering (DLS) analysis of 100 g/L oil-in-water emulsion. (d) Oil concentration measurement results along with FO

operation period.

In brief, the above results verify that placing the returning tubing tip of concentrate in feed tank above water level is successful to overcome the problem of oil/water stratification. And please note that all the data presented in this study are under the setting of placing the returning tubing tip above water level to maintain the oil/water mixture existing in homogenous emulsion form during testing period.

2.5. Characterization

Transmission electron microscopy (TEM, JEOL 2010-H) and atomic force microscopy (AFM, Park XE-100) were used to characterize the morphology of as-synthesized GO nanosheet. For the sample preparation, sonicated GO solution was dropped onto 400-mesh carbon coated copper grids or silicon wafer and then dried in room temperature for solvent evaporation. Field emission scanning electronic microscopy (FESEM, JEOL JSM 7600F) was used to characterize the structures of graphite oxide and membranes. All samples were coated by gold for 30 s using an EMITECH SC 7620 sputter coater. Membrane cross-sections were acquired by fracturing the samples immediately after flash-frozen in liquid nitrogen. X-ray diffraction (XRD) patterns were recorded using a Bruker AXS D8 Advance diffractormeter equipped with a Cu Kα radiation source. Attenuated total reflection-Fourier transform infrared spectroscopy (ATR-FTIR, Perkin Elmer 2000, ZnSe crystal method) was used to analyze the functional groups of membrane surface with samples freeze-dried overnight before scanned. Surface zeta-potential was

measured using streaming potential in the pH range 2~11 by a SurPASS electrokinetic analyzer (Anton Paar GmbH, Austria). Contact angles (CA) were determined on an optical goniometric equipment (AST VCA Optima) using sessile drop technique and reported as the average of at least 11 random measurements. Specifically, 3 μl DI water in air or 10 μl 1,2-dichloromethane under water were used as the probe liquid. And all CA data were recorded at the initial moment when probe liquid fully wet the solid surface. Dynamic light scattering (DLS, Mastersizer 2000) and optical microscopy (Olympus IX 71) were used to characterize oil droplet size distribution. Total organic carbon (TOC, Shimadzu TOC-VCSH) and chemical oxygen demand (COD, HACH method 8000 HR and ULR) were used to determine the content of total organics (including oil and surfactant). Ion chromatography (DIONEX ICS-1000) was used to analyze anion concentration, i.e. Cl^- in this study, while inductively coupled plasma-optical emission spectroscopy (ICP-OES, Perkin Elmer Optima 2000 DV) was used to analyze cation concentration, i.e. Mg^{2+} and Al^{3+} in this study.

3. Supplementary Figures and Tables

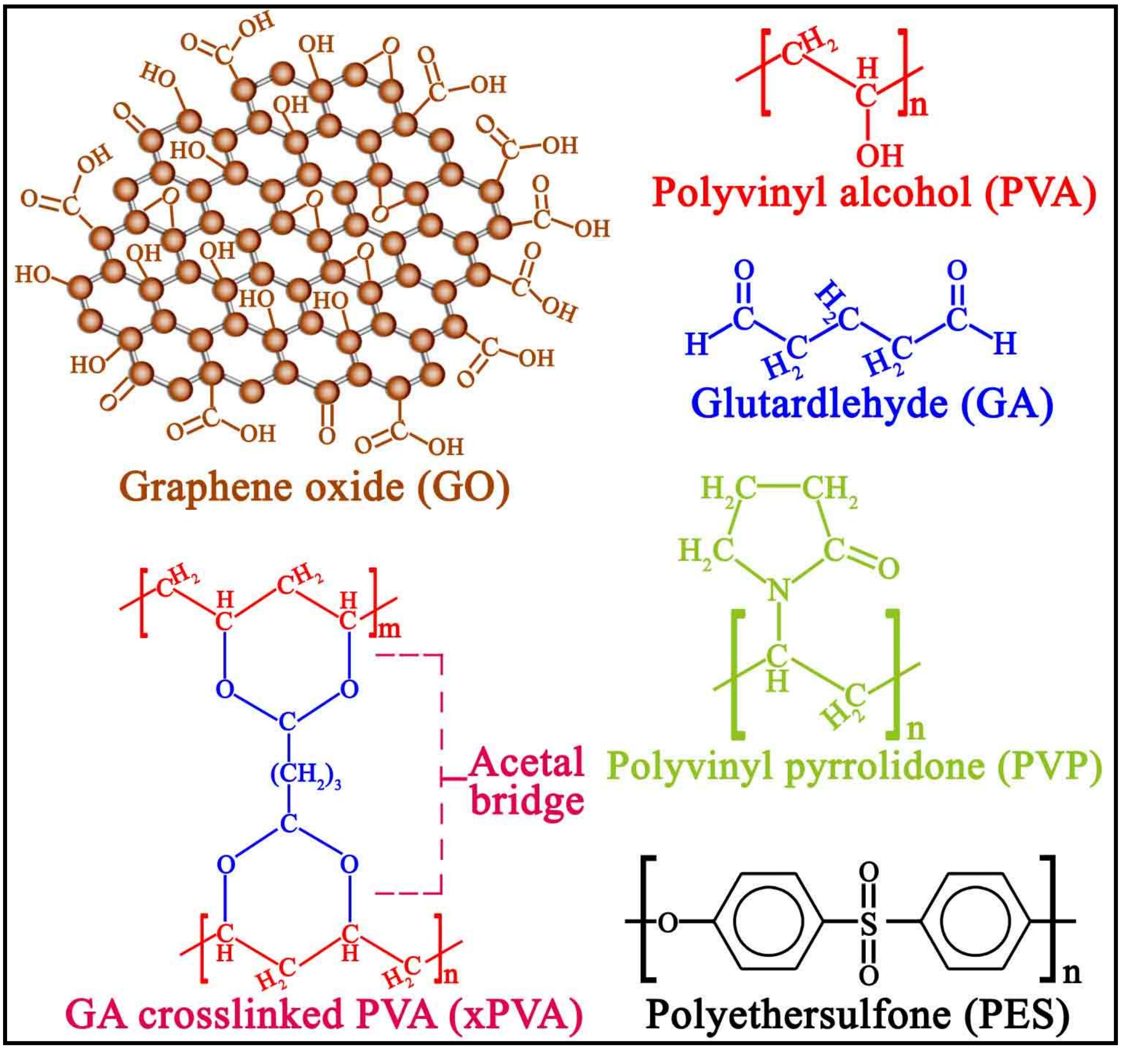

Figure S4. Molecular structures of individual chemicals associated with this study.

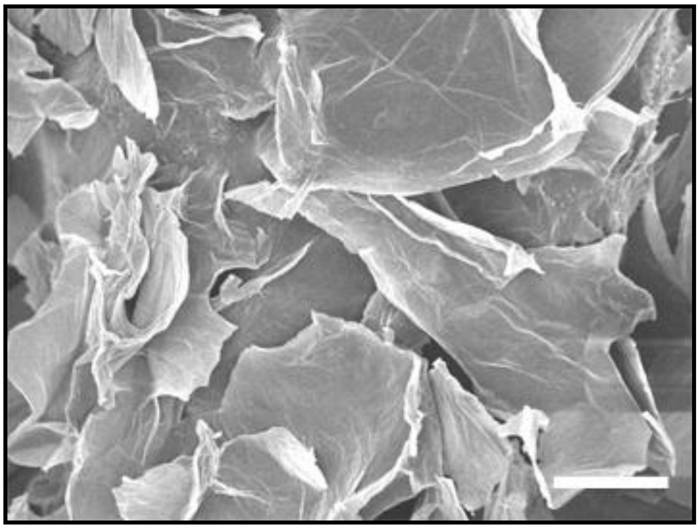

Figure S5. FESEM image of as-synthesized graphite oxide. The obtained graphite oxide exhibits disordered morphology as evidenced by plenty of wrinkles formed on its microplate surface, indicating the crystal structure of graphite is disturbed by intercalation and oxidation during synthetic process.

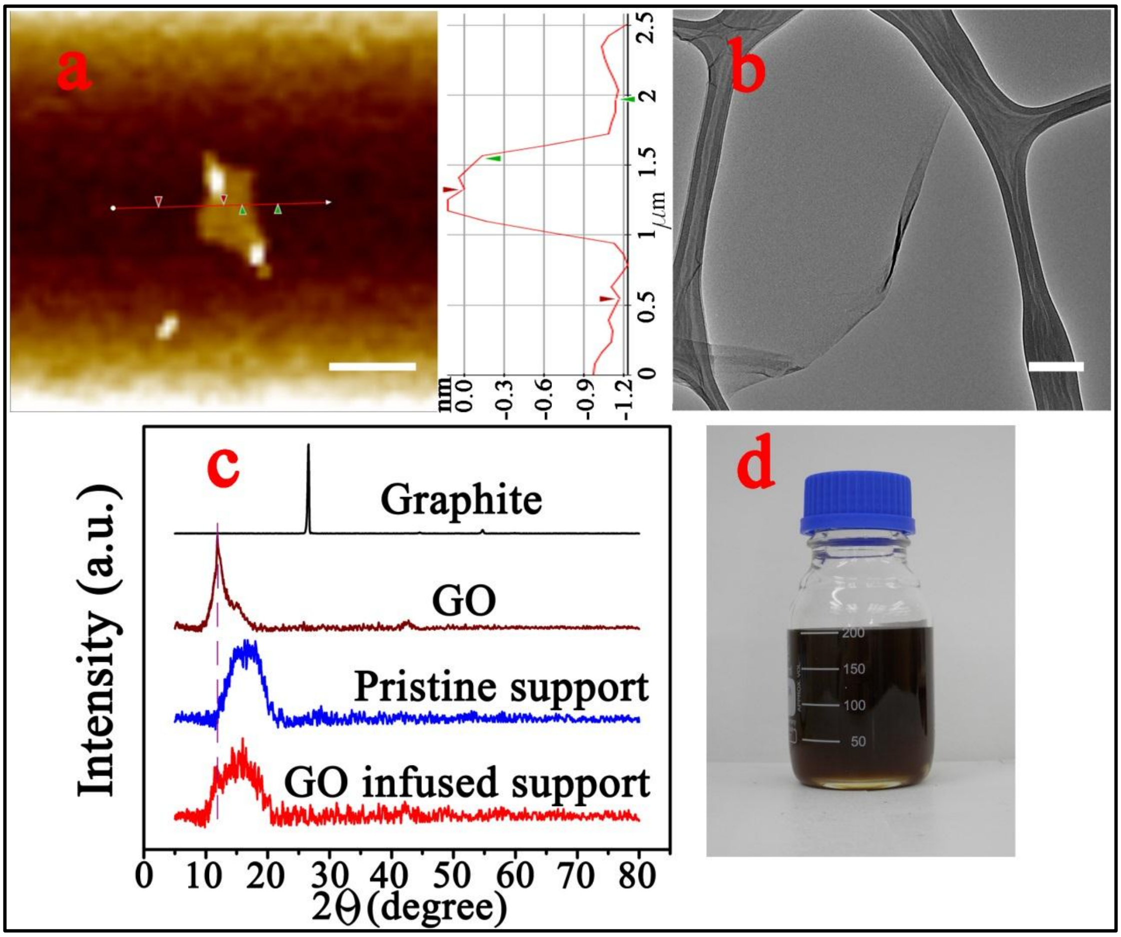

Figure S6. Characterization of GO nanosheet and its nanocomposite dope solution. (a) AFM image of a single graphene oxide sheet (scale bar, 1 μm). (b) TEM image of a single graphene oxide sheet (scale bar, 200 nm). (c) XRD patterns of graphite, GO, pristine polymeric support layer, and GO infused polymeric support layer, respectively. As marked by the dotted purple line, the 2θ peak at 11.8° on the spectrum of GO infused support layer confirms the incorporation of GO nanosheets into polymeric support layer matrix. (d) Optical photo of the nanocomposite (GO infused PES) dope solution, showing that GO nanosheets are uniformly dispersed to form a stable dope solution.

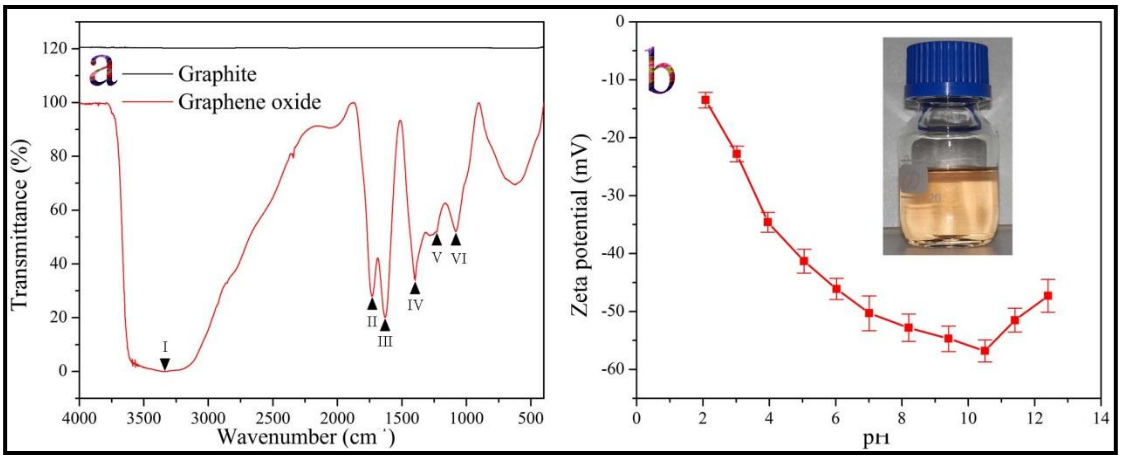

Figure S7. (a) FTIR spectra of graphite and GO, (b) zeta-potential of GO aqueous solution at different pH values. (a) The IR spectrum of graphite (black line) is featureless. In contrast, the IR spectrum of GO confirms that various functional groups are formed due to oxidation, with the band assignments elaborated in Table S2. (b) Inset figure is the optical photograph of GO aqueous solution (100 mg L^{-1}) showing that sonicating GO nanosheets in deionized water could obtain a homogenous solution in brown color. Zeta-potential test results indicate that the surface charge of GO sheet is highly pH sensitive: increasing OH^- concentration from $10^{-11.9}$ M to $10^{-3.5}$ M leads to the decrease of zeta-potential by 42 mV, mainly due to the deprotonation of carboxylic and phenolic hydroxyl groups on GO nanosheets.

Table S2. Band assignments of GO FTIR spectrum (as shown in Figure S7a).

IR band position (cm^{-1})	Marker	Assignments
3333	I	broad band from 3050 cm^{-1} to 3550 cm^{-1} indicating O-H stretching vibrations arisen from -OH groups of GO nanosheets and occluded/absorbed water molecules in GO layers
1732	II	the C=O stretching vibrations of –COOH groups
1630	III	the vibration resonance of adsorbed hydroxyl groups and unoxidized sp^2 C-C bonding in the carbon lattice
1398	IV	the –OH deformation of C-OH groups
1232	V	the stretching vibrations of C-O on epoxides (C-O-C)
1083	VI	the C-O stretching vibrations of –COOH groups

Table S3. Elaborate analysis of ATR-FTIR results (as shown in Figure 3a).

IR band position (cm^{-1})	Marker on Fig. 4a	Presence or absence on IR spectra				Functional groups assigned to
		Pristine support	GO infused support	PVA Hydrogel		
				300 nm	1μm	
3433	p	×	√	-	-	**O-H stretching of hydroxyl groups on GO**
3402	t	×	-	√	√	O-H stretching of hydroxyl groups on xPVA[5]
2947	u	×	×	√	√	C-H asymmetric stretching in alkyl (-CH_2-) groups of xPVA chain skeleton[6]
2873	v	×	×	√	√	C-H stretching of aldehyde group of xPVA[7]
1726	q	×	√	-	×	**C=O stretching of COOH on GO**[8]
1676	r	-	√	√	-	the stretching vibrations of primary amide group (-N-C=O) of PVP[9,10]
1578	a	√	√	√	×	C-H bond in the benzene ring of PES[11]
1487	b	√	√	√	×	C=C bond in the benzene ring of PES[12]
1440	w	×	×	√	√	Bending vibrations of C-H of xPVA[13]
1411	c	√	√	×	×	stretching vibrations of SO2 group of PES[14]
1382	x	×	×	√	√	C-OH stretching vibration of xPVA[15]
1348	y	×	×	×	√	C-OH stretching vibration of xPVA[15]
1325	d	√	√	√	×	asymmetric stretching of CSO2C of PES[14]
1300	e	√	√	√	×	asymmetric stretching of O=S=O of PES[16]
1244	f	√	√	√	-	stretching of aromatic ether of PES[17]
1153	g	√	√	√	×	symmetric stretching of O=S=O of PES[16]
1132	z	×	×	-	√	**C-O-C stretching vibrations of acetal bridge, characteristic peak of xPVA**.(**this study**)
1107	h	√	√	√	-	C-O bond of PES skeleton[11]
1072	i	√	√	-	×	C-O-C stretching of PES skeleton
1051	α	×	-	√	√	C-O stretching of acetal bridge of xPVA[18]
1050	s	×	√	-	-	C-O bond of COOH group of GO (**this study**)
1012	j	√	√	-	×	parasubstituted phenyl ethers of PES[14]
1002	β	×	×	-	√	O-C-O stretching of acetal bridge of xPVA[7]
881	γ	×	×	×	√	C-C stretching of xPVA skeleton[5]
873	k	√	√	√	×	parasubstituted benzene rings of PES[19]
839	l	√	√	√	×	out-of-plane C-H deformations of parasubstituted phenyls of PES[14]
833	δ	×	×	×	√	C-H bonding of the xPVA skeleton[5]
800	m	√	√	√	×	C-H out of plane bending[15]
719	n	√	√	√	×	C-S stretching vibrations of PES[14]
704	o	√	√	√	×	parasubstituted phenyls of PES

Note: IR Bands originated from pristine polymeric support layer (PES) are tabulated in grey background; IR bands originated from embedded GO nanosheets tabulated in blue background; IR bands originated from chemically-crosslinked hydrogel (xPVA)

selective layer are tabulated in pink background; IR bands originated from polymeric additive (PVP) are tabulated in green background. Symbol "√" refers to the presence of IR band; "×" refers to the absence or disappearance of IR band; "-"refers to the IR band in trace or not sharp form.

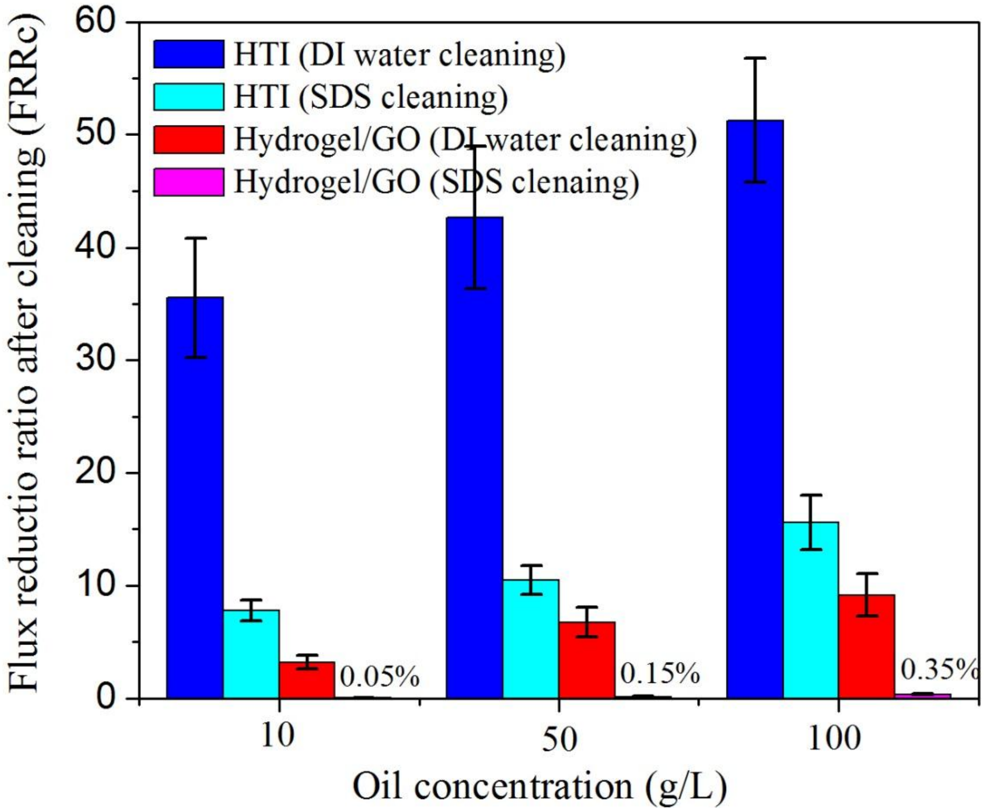

Figure S8. Flux reduction ratios (*FRRc*) after DI water or 0.2% SDS cleaning. Note that the pink columns, which refer to the *FRRc* values of Hydrogel/GO FO membrane after 0.2% SDS cleaning, appear approximately invisible in the diagram. This is because their values are much smaller ($\leq$ 0.35%) compared with other columns. Draw solution is 1.5 M Na_2SO_4 and feed solutions are surfactant-free oil-in-water emulsions.

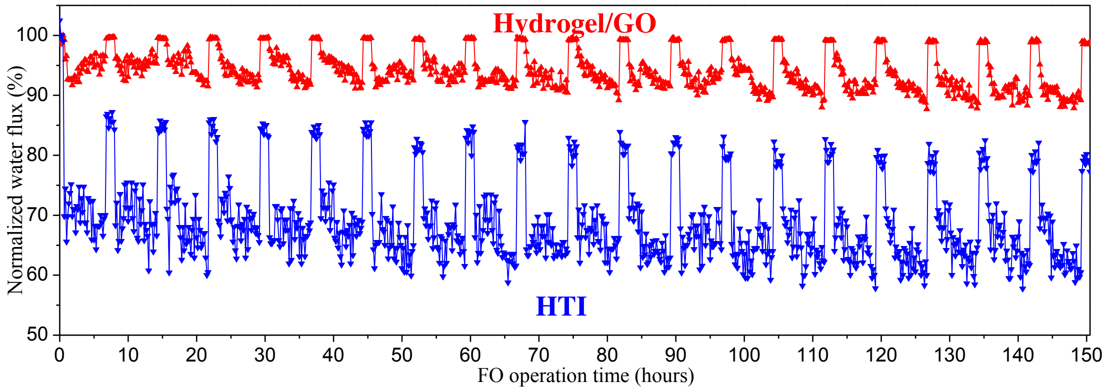

Figure S9. Long term operation results of synthesized Hydrogel/GO and commercial HTI FO membranes. Draw solution is 1.5 M Na_2SO_4. Feed solution is 25 g/L hexadecane-in-water emulsion with 0.05 surfactant/oil ratio. At the beginning of each FO cycle, a new batch of feed solution as well as draw solution is employed. This result shows that the highly antifouling advantage of Hydrogel/GO membrane over HTI membrane is durable in long term FO operation.

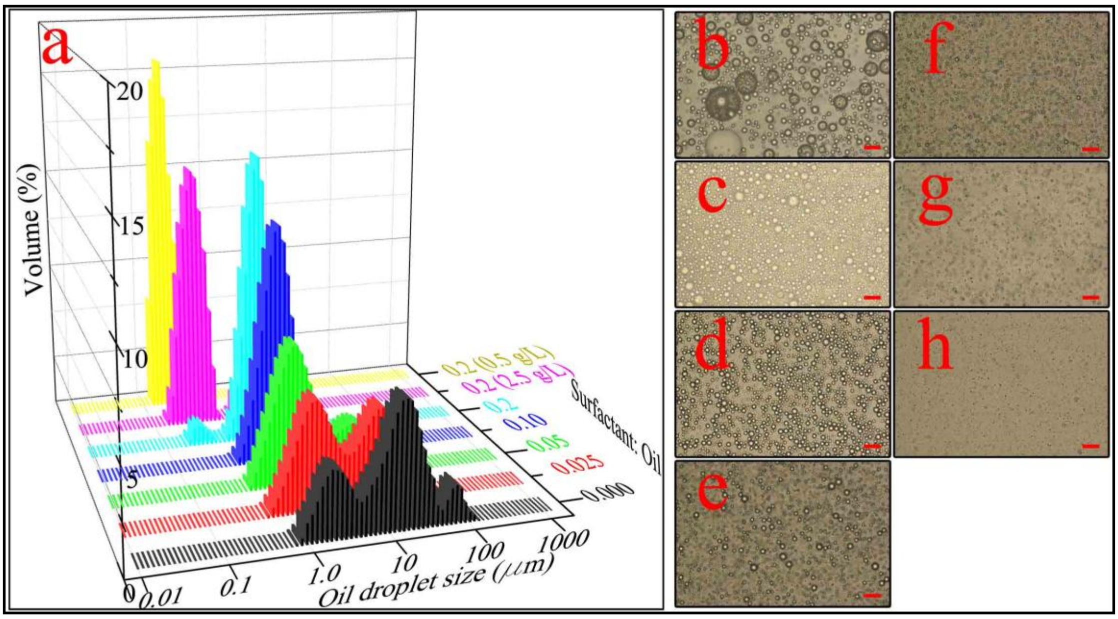

Figure S10. Oil droplet size distributions under different surfactant/oil ratios as well as different oil concentrations. (a) Dynamic light scattering (DLS) studies of oil droplet size distributions as a function of surfactant/oil ratios and oil concentrations. (b-h) Optical microscopic images of salinity-free oil-in-water emulsions (scale bar, 50 μm), wherein (b-f) the oil concentration is 50 g/L while the surfactant/oil ratios are 0.000, 0.025, 0.05, 0.1, 0.2, respectively, (g-h) the surfactant/oil ratio is 0.2 while the oil concentrations are 2.5 and 0.5 g/L, respectively. The details of oil droplet size distributions are elaborated in Table S4.

Table S4. Details of oil droplet size distributions (as shown in Figure S10).

Surfactant/oil ratio	Oil concentration (g/L)	Peak positions on droplet size distribution	Optical Microscopy
0.000	50	a minor peak at 69.2 μm (volume 1.84%),<br>a major peak at 13.2 μm (volume 6.04%),<br>a minor peak at 1.93 μm (volume 3.30%).	Fig. S10b
0.025	50	a major peak at 15.1 μm (volume 4.59%),<br>a major peak at 2.19 μm (volume 5.27%).	Fig. S10c
0.050	50	a minor peak at 11.5 μm (volume 2.70%),<br>a major peak at 1.91 μm (volume 6.79%).	Fig. S10d
0.10	50	a major peak at 2.18 μm (volume 11.59%).	Fig. S10e
0.20	50	a major peak at 1.90 μm (volume 14.26%),<br>a minor peak at 275 nm (volume 1.03%)	Fig. S10f
0.20	2.5	a minor peak at 1.90 μm (volume 1.6%),<br>a major peak at 363 nm (volume 13.11%).	Fig. S10g
0.20	0.5	a main peak at 209 nm (volume 18.08%).	Fig. S10h

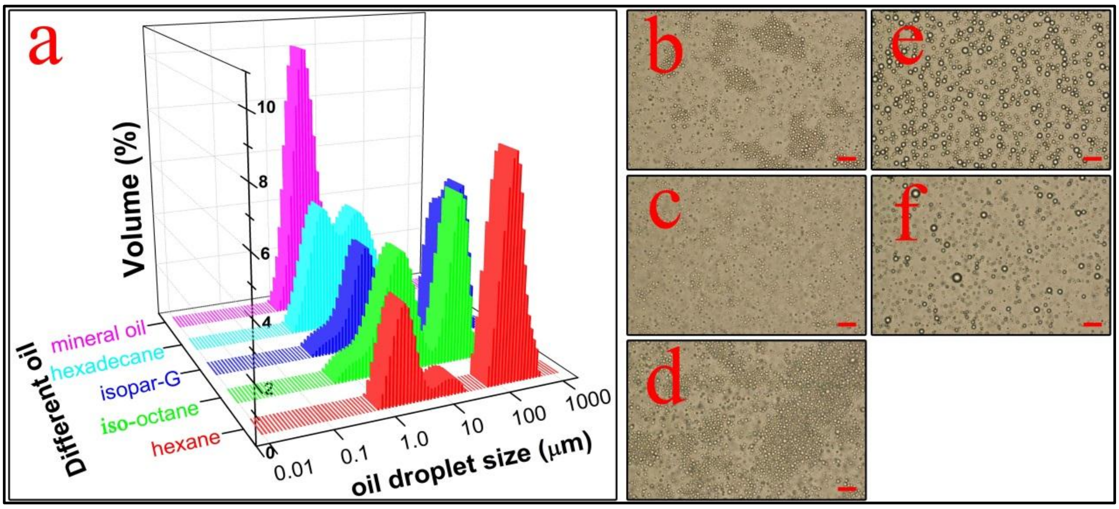

Figure S11. Oil droplet size distributions of different kinds of oil. (a) Dynamic light scattering (DLS) studies of oil droplet size distributions under different kinds of oil. (b-f) Optical microscopic images of salinity-free emulsions prepared from different kinds of oils (scale bar, 50 μm; the oil concentration is 25 g/L and the surfactant/oil ratios is 0.05), wherein (b) hexane, (c) iso-octane, (d) isopar-G, (e) hexadecane, and (f) mineral oil, respectively. The details of oil droplet size distributions are elaborated in Table S5.

Table S5. Details of oil droplet size distributions (as shown in in Figure S11).

Different oil	Oil concentration<br>(Surfactant/oil ratio)	Peak positions on droplet size distribution	Optical Microscopy
n-hexane	25 g/L (0.05)	a major peak at 138.0 μm (volume 7.89%),<br>a minor peak at 11.48 μm (volume 0.60%),<br>a major peak at 1.91 μm (volume 3.44%).	Fig. S11b
Iso-octane<br>(Trimethylpentane)	25 g/L (0.05)	a major peak at 69.2 μm (volume 5.98%),<br>a major peak at 5.75 μm (volume 4.27%).	Fig. S11c
Isopar-G	25 g/L (0.05)	a major peak at 316 μm (volume 5.57%)<br>a major peak at 158 μm (volume 4.91%),<br>a major peak at 4.37 μm (volume 3.75%).	Fig. S11d
n-hexadecane	25 g/L (0.05)	a major peak at 10.0 μm (volume 4.35%),<br>a major peak at 2.19 μm (volume 4.63%).	Fig. S11e
Mineral oil	25 g/L (0.05)	a major peak at 2.51 μm (volume 10.80%).	Fig. S11f

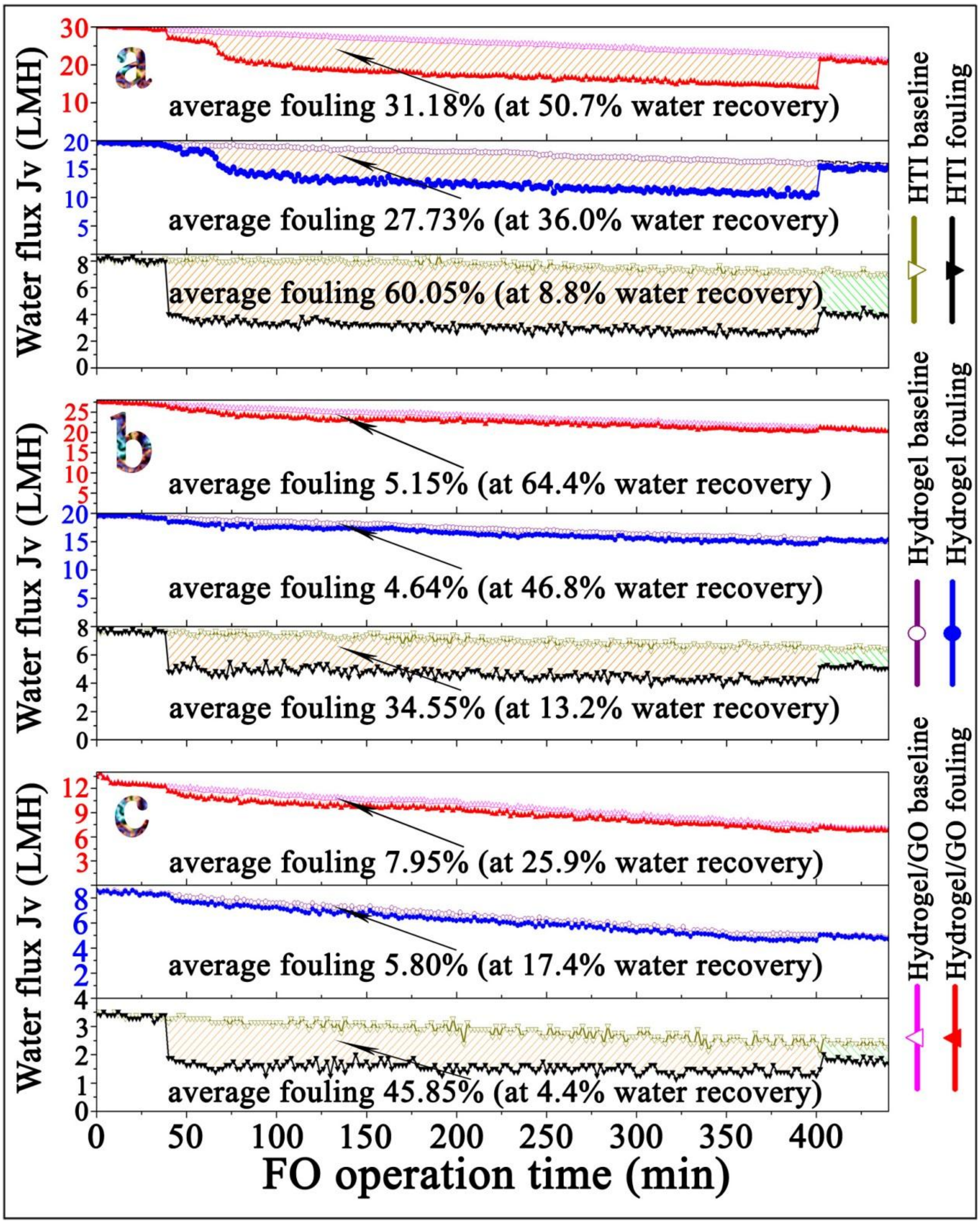

Figure S12. Systematic investigation on Jv-time functions of both salinity-free emulsions and shale gas wastewater. Draw solution is 1.5 M Na_2SO_4. (a) Feed solution is DI water for "baseline running", while surfactant-free hexadecane-in-water emulsion with 25 g/L oil concentration and 0 g/L TDS for "fouling running". (b) Feed solution is DI water for "baseline running", while surfactant-stabilized hexadecane-in-water emulsion with 25 g/L oil concentration, 0.05 surfactant/oil ratio and 0 g/L TDS for "fouling running". (c) Feed solution is 156 g/L TDS in DI water for "baseline running", while surfactant-stabilized hexadecane-in-water emulsion with 25 g/L oil concentration, 0.05 surfactant/oil ratio and 156 g/L TDS for "fouling running", which is designed for simulated shale gas wastewater treatment. For each "fouling

running”, oil-in-water emulsion is used as the feed solution from 41th min to 400th min; and the shadow area indicates the average flux reduction ratio at given operation time.

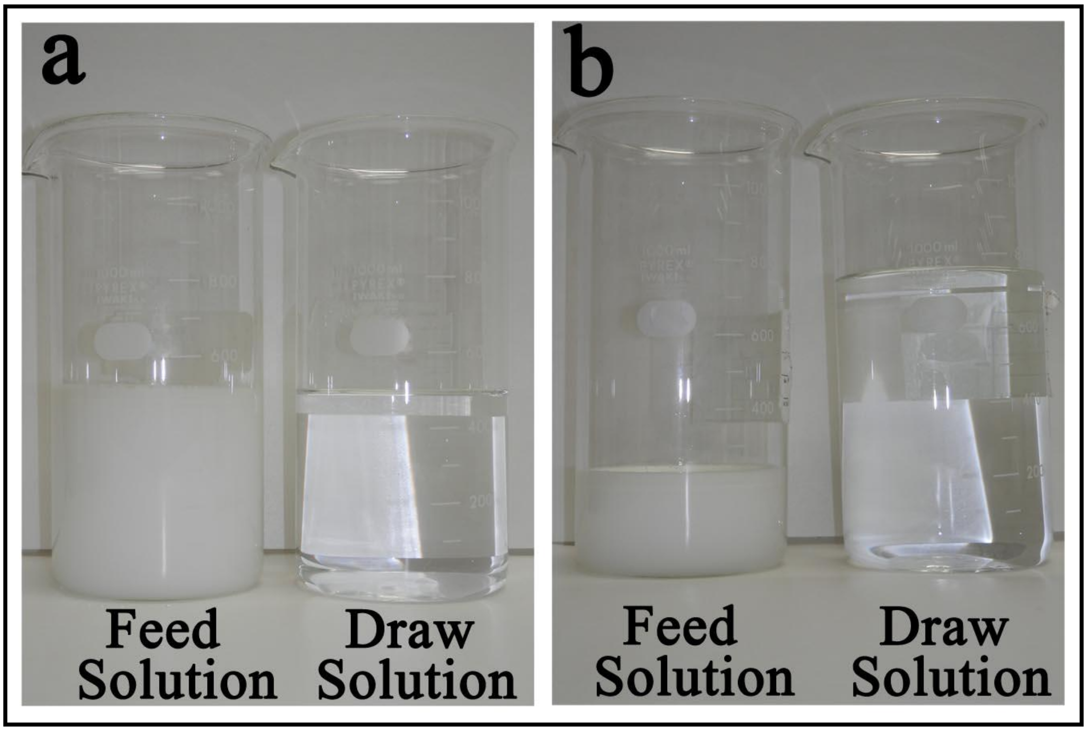

Figure S13. Optical photographs of feed and draw solutions for simultaneously deoilling and desalting shale gas wastewater by Hydrogel/GO FO membrane. Draw solution is 1.5 M Na_2SO_4. Feed solution is surfactant-stabilized hexadecane-in-water emulsion with 25 g/L oil concentration, 0.05 surfactant/oil ratio and 156 g/L TDS, which is used as simulated shale gas wastewater. (a) Before the "oil-fouling running". (b) At the end of "oil-fouling running".

Table S6. Water quality analysis results of feed and draw solutions at the end of "oil-fouling stage" (400th min) for simulated shale gas wastewater treatment.

Parameter	HTI Feed water	HTI Draw solution	Hydrogel/GO Feed water	Hydrogel/GO Draw solution
COD (mg/L)	75,502 ± 4,314	0.5 ± 0.2	120,236 ± 6,010	0.5 ± 0.1
TOC (mg/L)	9,419 ± 566	0.30 ± 0.05	15,283 ± 928	0.18 ± 0.04
$(Al^{3+})_{total}$ (mg/L)	16,814 ± 2,068	0.53 ± 0.06	20,335 ± 2,745	1.02 ± 0.14
$(Mg^{2+})_{total}$ (mg/L)	7,853 ± 709	0.88 ± 0.13	9,033 ± 838	3.75 ± 0.53
Cl^- (mg/L)	10,973 ± 924	75.5 ± 9.1	12,590 ± 1,217	516 ± 42
Turbidity (NTU)	>99,999	0.125±0.02	>99,999	0.130±0.015
Color (hazen)	1,780 ± 43	0.000	2,135 ± 65	0.000
Conductivity(mS/cm)	54.5 ± 1.3	117.3 ±1.1	57.8 ± 1.4	112.5 ± 2.5
Temperature (°C)	23.5 ± 0.3	23.5 ± 0.3	23.5 ± 0.3	23.5 ± 0.3

Note: Draw solution is 1.5 M Na_2SO_4. Feed solution is surfactant-stabilized hexadecane-in-water emulsion with 25 g/L oil concentration, 0.05 surfactant/oil ratio and 156 g/L TDS, which is used as simulated shale gas wastewater. Because Hydrogel/GO membrane achieves much higher water recovery than HTI membrane at the given operation time, the concentration of pollutant in the draw solution of Hydrogel/GO membrane is higher than that of HTI membrane at the end of "oil-fouling" stage.

4. Supplementary Discussions

4.1 Characterization of GO nanosheet and its nanocomposite dope solution (as shown in Figure S6-S7).

Figure S6 shows that graphene oxide sheets in nanometer (nm) scale thickness were successfully prepared through exfoliating as-synthesized graphite oxide (Figure S5). AFM image indicates that a single GO sheet is ~1.2 nm in thickness (Figure S6a), which is slightly thicker than graphene monolayer[20]. The nanometer scale thickness renders GO monolayer approximately transparent in TEM image (Figure S6b), though its lateral sizes are in micrometer scale. XRD patterns indicate that the interlayer spacing of GO sheets is increased to 7.44 Å (2θ peak at 11.8°) as the result of intercalation and oxidation (Figure S6c). And FTIR spectra confirm the existence of various oxygen-containing functional groups, *e.g*. hydroxyl (IR peak 3333 cm^{-1} and 1398 cm^{-1}), carboxyl (IR peak 1732 cm^{-1}) and epoxy (IR peak 1232 cm^{-1}) groups, on GO nanosheets (Figure S7a and Table S2). Moreover, zeta-potential characterization results reveal that ionization of these oxygenic functional groups leads to negatively charged GO surface in a wide pH range, which is essential to maintain GO dispersion stable by electrostatic repulsion effect (Figure S7b).

4.2 Elaborate analysis of ATR-FTIR spectra (as shown in Figure 3a).

As shown in Figure 3a and Table S3, the bands on the ATR-FTIR spectrum of pristine PES support layer at 1578 cm^{-1} (peak a) and 1487 cm^{-1} (peak b) are associated with the vibrations of C-H bond and C=C bond respectively in the benzene ring of polymeric skeleton. The band observed at 1325 cm^{-1} (peak d) has been assigned to the asymmetric stretching of CSO_2C in the polymeric backbone. The bands at 1300 cm^{-1}

(peak e) and 1153 cm^{-1} (peak g) are attributed the asymmetric and symmetric stretching vibrations of O=S=O groups in PES skeleton, respectively. And the bands at 1244 cm^{-1} (peak f) and 1107 cm^{-1} (peak h) are related to C-O vibrations of the aromatic ether linkage in the backbone. The above seven peaks are the characteristic bands of PES, which emerge clearly on the IR spectra of both pristine and GO infused polymeric support layers, get weakened on Hydrogel/GO FO membrane (300 nm hydrogel selective layer thickness) spectrum because chemically-crosslinked hydrogel (xPVA) selective layer is coated at relatively thin thickness, and eventually disappear with few traces left as selective layer thickness increased to 1 μm.

On the ATR-FTIR spectrum of GO infused polymeric support layer, three new bands are observed compared with the IR spectrum of pristine polymeric support layer: a wide band centered at 3433 cm^{-1} (peak p) due to the O-H stretching vibrations of hydroxyl groups, the band at 1726 cm^{-1} (peak q) due to the C=O stretching vibration of carboxyl groups, and the band at 1050 cm^{-1} (peak s) due to the C-O stretching vibration of carboxyl and epoxy groups. These three IR bands confirm that the infused GO nanosheets equip support layer top surface with various oxygenic functional groups.

The ATR-FTIR analysis for Hydrogel/GO FO membrane with selective layer (xPVA) thickness of 100 nm has been conducted. However, the result appears to be misleading because its IR spectrum is almost identical to that of GO infused

polymeric support layer. This is ascribed to three reasons. Firstly, ATR-FTIR technique can probe chemical information of solid surface at a depth around 1 μm (depending on the surface compactness). So the support layer beneath an ultrathin functional layer can be detected in many cases. Secondly, the spectra of GO infused polymeric support layer and hydrogel selective layer (xPVA) are overlapping each other in terms of characteristic bands. Thirdly, the specific response of GO infused polymeric support layer in ATR-FTIR scanning is stronger than that of hydrogel selective layer. As a result, when hydrogel selective layer (xPVA) is not thick enough, though excessive thickness of selective layer is not favorable for membrane separation performance, its IR signal would be completely veiled by that of GO infused polymeric support layer underneath. Under this circumstance, we purposely prepared Hydrogel/GO FO membrane samples with selective layer thickness of 300 nm and 1 μm, whose ATR-FTIR spectra signify the transition from support layer spectrum to selective layer spectrum (300 nm thickness), and the spectrum fully featured by hydrogel selective layer (1μm thickness), respectively.

And in order to avoid any confusion, the IR bands originated from chemically-crosslinked hydrogel are marked only on the spectrum of 1 μm thickness with red symbols "t-z" and "α-δ" as shown in Figure 3a. In detail, the IR band at 3402 cm^{-1} (peak t) is the O-H stretching of unreacted hydroxyl groups on PVA chains. The IR band at 2947 cm^{-1} (peak u) is associated with the C-H asymmetric stretching of alkyl groups ($-CH_2-$) in the xPVA skeleton. And the "paw-type" band cluster exists

between 1000 cm^{-1} and 1150 cm^{-1} are assigned to the C-O-C (1132 cm^{-1}, peak z), C-O (1150 cm^{-1}, peak α) and O-C-O (1002 cm^{-1}, peak β) stretching vibrations of acetal bridges (see molecular structure of acetal bridge in Figure S4), which are formed by aldolization of aldehyde groups (-CHO) of glutaraldehyde with hydroxyl groups (-OH) of PVA. These five peaks are the characteristic bands of glutaraldehyde crosslinked PVA hydrogel selective layer.

4.3 Elaborate analysis of the correlations between membrane fouling and oil droplet size distribution of emulsion (as shown in Figure 5i-j).

To analyze particle size distribution from the perspective of statistics, three interdependent indicators namely d_{10}, d_{50} and d_{90} are usually calculated, wherein d_{50} refers to the particle (oil droplet) diameter at the cumulative mass proportion of 50%. Therefore d_{50} can be regarded as the average particle size to represent the distribution in a simplified way. Here, mathematical fittings between FRR_f and d_{50} were conducted to study the influence of oil droplet size distribution on membrane fouling extent.

Firstly, the potential link between FRR_f and d_{50} under emulsions prepared from the same kind of oil (vegetable oil) is investigated, and thus the chemical affinity with oil is unchanged for a certain membrane. Herein, the emulsions with different d_{50} were prepared through adjusting surfactant/oil ratios as well as oil concentrations, as shown in Figure S10. In detail, the d_{50} of 50 g/L emulsion is reduced from 10.5 μm to 2.55 μm as surfactant/oil ratio increased from 0.00 to 0.05. However, further increasing surfactant/oil ratio to 0.2 only reduces d_{50} to 1.76 μm. This indicates that it's not

effective to control major size distribution of oil droplets below 1.0 μm only through increasing surfactant concentration, because the oil concentration is too high to avoid the agglomeration of submicrometer sized droplets. Therefore, submicrometer sized emulsions were purposely prepared by reducing the oil concentration to 2.5 and 0.5 g/L with surfactant/oil ratio kept as 0.2. Correspondingly, Figure 5i indicates that positive correlations between FRR_f and d_{50} exist for both Hydrogel/GO and HTI FO membranes. And it's evident that the data points on Figure 5i can be grouped into three clusters, which refer to surfactant-free emulsions, surfactant-stabilized microsized emulsions and surfactant-stabilized nanosized emulsions, respectively. Strong linear correlation between d_{50} and FRR_f is found within each cluster separately. However, the slope of linear fitting in each region cannot be extrapolated to another region. More importantly, the FRR_f -d_{50} curve slope of HTI membrane changes in much greater extents from one region to the next region, compared with that of Hydrogel/GO membrane. This indicates that the fouling of underwater oleophilic surface is highly dependent on oil droplet size distribution.

Secondly, the potential link between FRR_f and d_{50} for emulsions prepared from different petroleum oils is investigated, with oil concentration and surfactant/oil ratio fixed as 25 g/L and 0.05, respectively. The corresponding oil droplet size distribution results are shown in Figure S11. It's obvious that the data points on Figure 5j can be grouped into two separate clusters based on the dispersibility of oil for both Hydrogel/GO and HTI membranes. One is named as "well-dispersed cluster"

referring to oil droplets remain detached without aggregation, while the other is named as “aggregates formed cluster” referring to macroaggregates of 100~500 μm are formed in emulsions. Within each cluster, linear correlation between FRR_f and d_{50} is established. However, regarding the correlation throughout the two clusters, HTI membrane and Hydrogel/GO membrane exhibits different trends. Interestingly, the FRR_f of HTI FO membrane establishes the order as: isopar-G > hexane > iso-octane (2,2,4-trimethypentane) > hexadecane > mineral oil, which is basically in conformity with the order of $d_{50.}$ However, such conformity does not exist for Hydrogel/GO membrane. For example, iso-octane (2,2,4-trimethylpentane) belongs to “well-dispersed” cluster because its oil droplets remains detached without aggregation in emulsion. But the FRR_f of iso-octane for Hydrogel/GO membrane approaches or even exceeds those belongs to “aggregates formed cluster”. This result indicates that factors other than oil droplet size (e.g. chemical affinity between oil and surface as discussed previously) might also play significant roles in membrane fouling.

4.4 Elaborate analysis of Jv-time functions under various emulsions (as shown in Figure S12).

There are three additional points to be noted for Figure S12. The first point is that HTI FO membrane suffers a sudden drop of water flux by 35%~60% once being fed with oil-in-water emulsions, indicating its underwater oleophilic property. In contrast, the J_V values of as-synthesized FO membranes take a ~40 min slow decline at much smaller rates before reaching stabilization, indicating their superior fouling-resistances. The second point is that the synthesized FO membrane with GO

infused polymeric support layer is slightly higher than that with pristine one in terms of FRR_f under each feed solution. This is probably because the incorporation of GO nanosheets renders the topography of polymeric support layer to be rougher, and hence increases the surface roughness of subsequently coated hydrogel selective layer. And the third point is that as-synthesized FO membranes surpass HTI FO membrane in terms of water recovery under each feed solution, mainly because of their higher water fluxes and lower membrane fouling tendencies.

Reference of Supplementary Information

1 McCutcheon, J. R. & Elimelech, M. Modeling water flux in forward osmosis: implications for improved membrane design. *Aiche J.* **53**, 1736-1744 (2007).

2 Cath, T. Y., Childress, A. E. & Elimelech, M. Forward osmosis: principles, applications, and recent developments. *J. Membr. Sci.* **281**, 70-87 (2006).

3 Dabelstein, W., Reglitzky, A., Schütze, A. & Reders, K. Automotive fuels. *Ullmann's Encyclopedia of Industrial Chemistry* (2007).

4 Bowen, S. E. & Balster, R. L. The effects of inhaled isoparaffins on locomotor activity and operant performance in mice. *Pharmacol. Biochem. Behav.* **61**, 271-280 (1998).

5 de Oliveira, A. A. R., Gomide, V. S., Leite, M. D., Mansur, H. S. & Pereira, M. D. Effect of Polyvinyl Alcohol Content and After Synthesis Neutralization on Structure, Mechanical Properties and Cytotoxicity of Sol-Gel Derived Hybrid Foams. *Mater. Res.-Ibero-am. J. Mater.* **12**, 239-244 (2009).

6 Mansur, H. S., Orefice, R. L. & Mansur, A. A. P. Characterization of poly(vinyl alcohol)/poly(ethylene glycol) hydrogels and PVA-derived hybrids by small-angle X-ray scattering and FTIR spectroscopy. *Polymer* **45**, 7193-7202 (2004).

7 Destaye, A. G., Lin, C. K. & Lee, C. K. Glutaraldehyde Vapor Cross-linked Nanofibrous PVA Mat with in Situ Formed Silver Nanoparticles. *ACS Appl. Mater. Interfaces* **5**, 4745-4752 (2013).

8 Ganesh, B. M., Isloor, A. M. & Ismail, A. F. Enhanced hydrophilicity and salt rejection study of graphene oxide-polysulfone mixed matrix membrane. *Desalination* **313**, 199-207 (2013).

9 Li, J. *et al.* One-Pot Synthesized Poly(vinyl pyrrolidone-co-methyl methacrylate-co-acrylic acid) Blended with Poly(ether sulfone) to Prepare Blood-Compatible Membranes. *J. Appl. Polym. Sci.* **130**, 4284-4298 (2013).

10 Vatsha, B., Ngila, J. C. & Moutloali, R. M. Preparation of antifouling polyvinylpyrrolidone (PVP 40K) modified polyethersulfone (PES) ultrafiltration (UF) membrane for water purification. *Phys. Chem. Earth* **67-69**, 125-131 (2014).

11 Wang, Y. Q. *et al.* Improved permeation performance of Pluronic F127-polyethersulfone blend ultrafiltration membranes. *J. Membr. Sci.* **282**, 44-51 (2006).

12 Susanto, H. & Ulbricht, M. Characteristics, performance and stability of polyethersulfone ultrafiltration membranes prepared by phase separation method using different macromolecular additives. *J. Membr. Sci.* **327**, 125-135 (2009).

13 Mansur, H. S., Sadahira, C. M., Souza, A. N. & Mansur, A. A. P. FTIR spectroscopy characterization of poly (vinyl alcohol) hydrogel with different hydrolysis degree and chemically crosslinked with glutaraldehyde. *Mater. Sci. Eng. C-Biomimetic Supramol. Syst.* **28**, 539-548 (2008).

14 Ahmed, I., Idris, A., Noordin, M. Y. & Rajput, R. High Performance Ultrafiltration Membranes Prepared by the Application of Modified Microwave Irradiation Technique. *Ind. Eng. Chem. Res.* **50**, 2272-2283 (2011).

15 Shaikh, R. P., Kumar, P., Choonara, Y. E., du Toit, L. C. & Pillay, V. Crosslinked electrospun PVA nanofibrous membranes: elucidation of their physicochemical, physicomechanical and molecular disposition. *Biofabrication* **4** (2012).

16 Li, J. Y., Oshima, A., Miura, T. & Washio, M. Preparation of the crosslinked polyethersulfone films by high-temperature electron-beam irradiation. *Polym. Degrad. Stabil.* **91**, 2867-2873 (2006).

17 Phao, N., Nxumalo, E. N., Mamba, B. B. & Mhlanga, S. D. A nitrogen-doped carbon nanotube enhanced polyethersulfone membrane system for water treatment. *Phys. Chem. Earth* **66**, 148-156 (2013).

18 Fang, X. *et al.* Facile immobilization of gold nanoparticles into electrospun polyethyleneimine/polyvinyl alcohol nanofibers for catalytic applications. *J. Mater. Chem.* **21**, 4493-4501 (2011).

19 Prasad, S. G., De, A. & De, U. Structural and optical investigations of radiation damage in transparent PET polymer films. *International Journal of Spectroscopy* **2011** (2011).

20 Novoselov, K. S. *et al.* Two-dimensional atomic crystals. *Proc. Natl. Acad. Sci. U. S. A.* **102**, 10451-10453 (2005).
